# Supplementary material for: Dual Inhibition of the Lactate Transporters MCT1 and MCT4 Is Synthetic Lethal with Metformin due to NAD+ Depletion in Cancer Cells
Source: Cell Rep. 2018 Dec 11;25(11):3047–3058.e4. doi: 10.1016/j.celrep.2018.11.043 (PMC6302548; doi:10.1016/j.celrep.2018.11.043)
Supplement: Document S1. Figures S1–S6 [file mmc1.pdf]

Cell Reports, Volume *25*

Supplemental Information

Dual Inhibition of the Lactate Transporters MCT1 and MCT4 Is Synthetic Lethal with Metformin due to NAD+ Depletion in Cancer Cells

Don Benjamin, Dimitri Robay, Sravanth K. Hindupur, Jens Pohlmann, Marco Colombi, Mahmoud Y. El-Shemerly, Sauveur-Michel Maira, Christoph Moroni, Heidi A. Lane, and Michael N. Hall

Figure S1

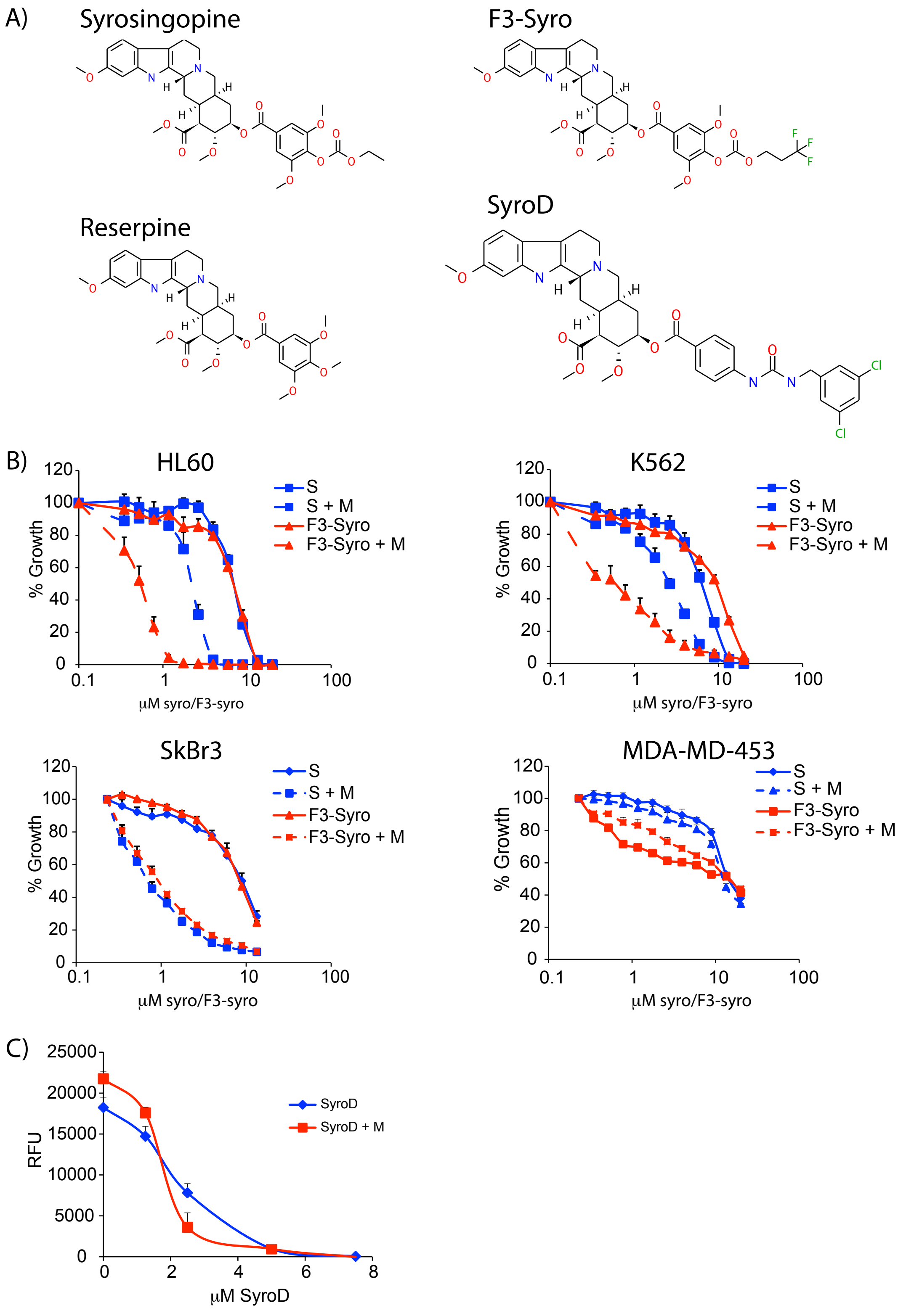

Figure S1. Relative potency of syrosingopine derivatives for synthetic lethality with metformin. Related to Figures 1 and 2. (A) Structures of the parent compound reserpine and its derivatives syrosingopine and F3-syro. SyroD is inactive in eliciting synthetic lethality with metformin. **(B)** Synthetic lethality between syrosingopine (S) or F3-syro (F3-syro) and metformin (M) in the human cancer lines HL60, K562, SkBr3 and MDA-MB-453. **(C)** Proliferation of HL60 cells titrated with SyroD in the presence/absence of metformin. Survival was measured after 3 days of drug treatment. All data points were measured in triplicate, represented as mean +/- SEM.

Fig S2

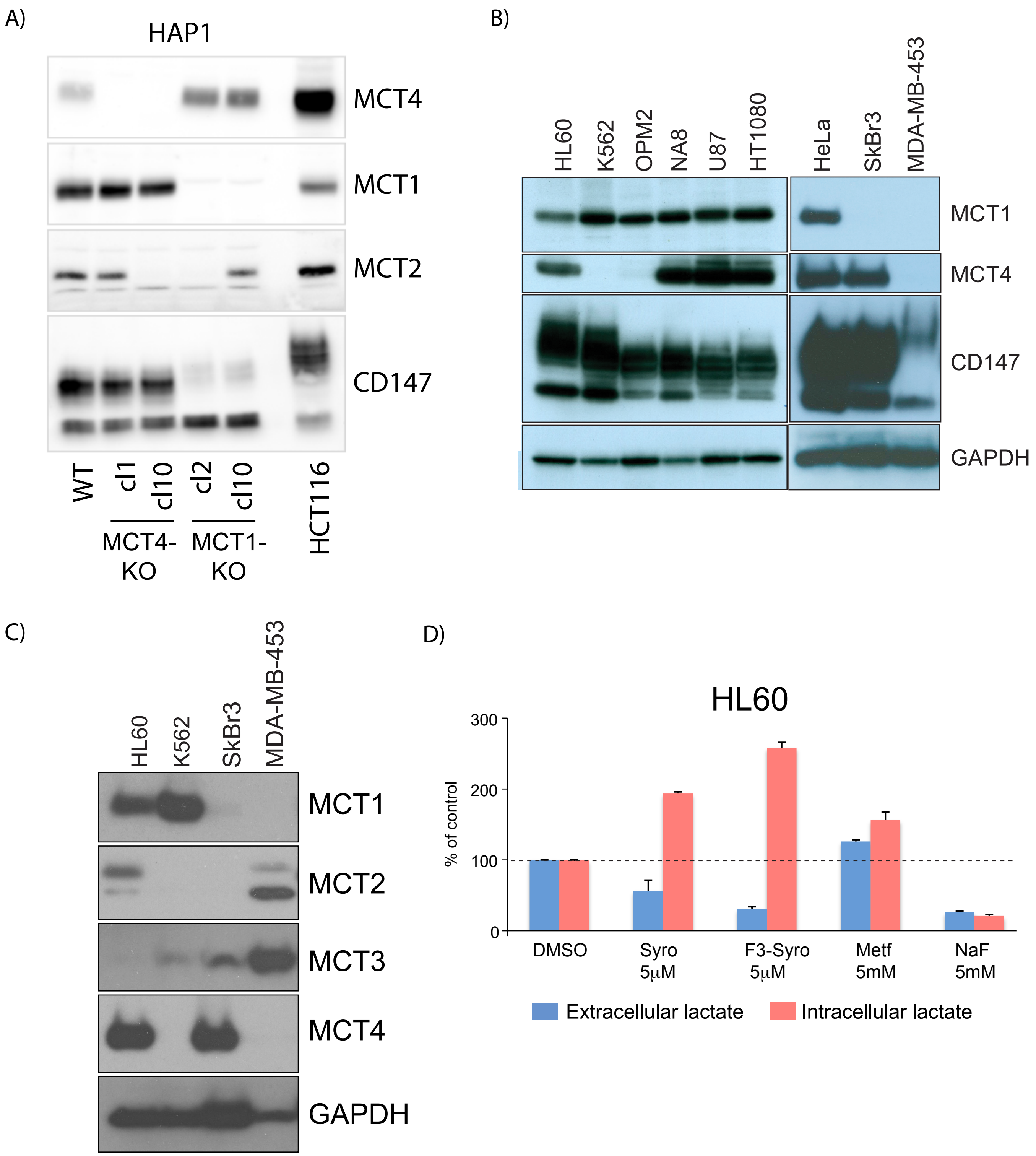

Figure S2. MCT isoform expression in HAP1 MCT-knockout panel and human cancer cell lines. Related to Figures 2, 3 and 5. (A) Immunoblot of HAP1 cells deleted for MCT1 (clones cl2 and cl10) or MCT4 (clones cl1 and cl10). **(B)** Immunoblot for MCT1 and MCT4 expression and their chaperone CD147 in various human cancer cell lines. HL60, K562, SkBr3 and MDA-MB-453 were selected to compose a cell panel. **(C)** Immunoblot of MCT1-4 in the cell panel. **(D)** Extra- and intra-cellular lactate levels in HL60 cells after 6 hours of treatment with syrosingopine, F3-syro, metformin and the glycolytic inhibitor NaF. All data points were measured in duplicate, bars are mean +/- SEM.

Figure S3

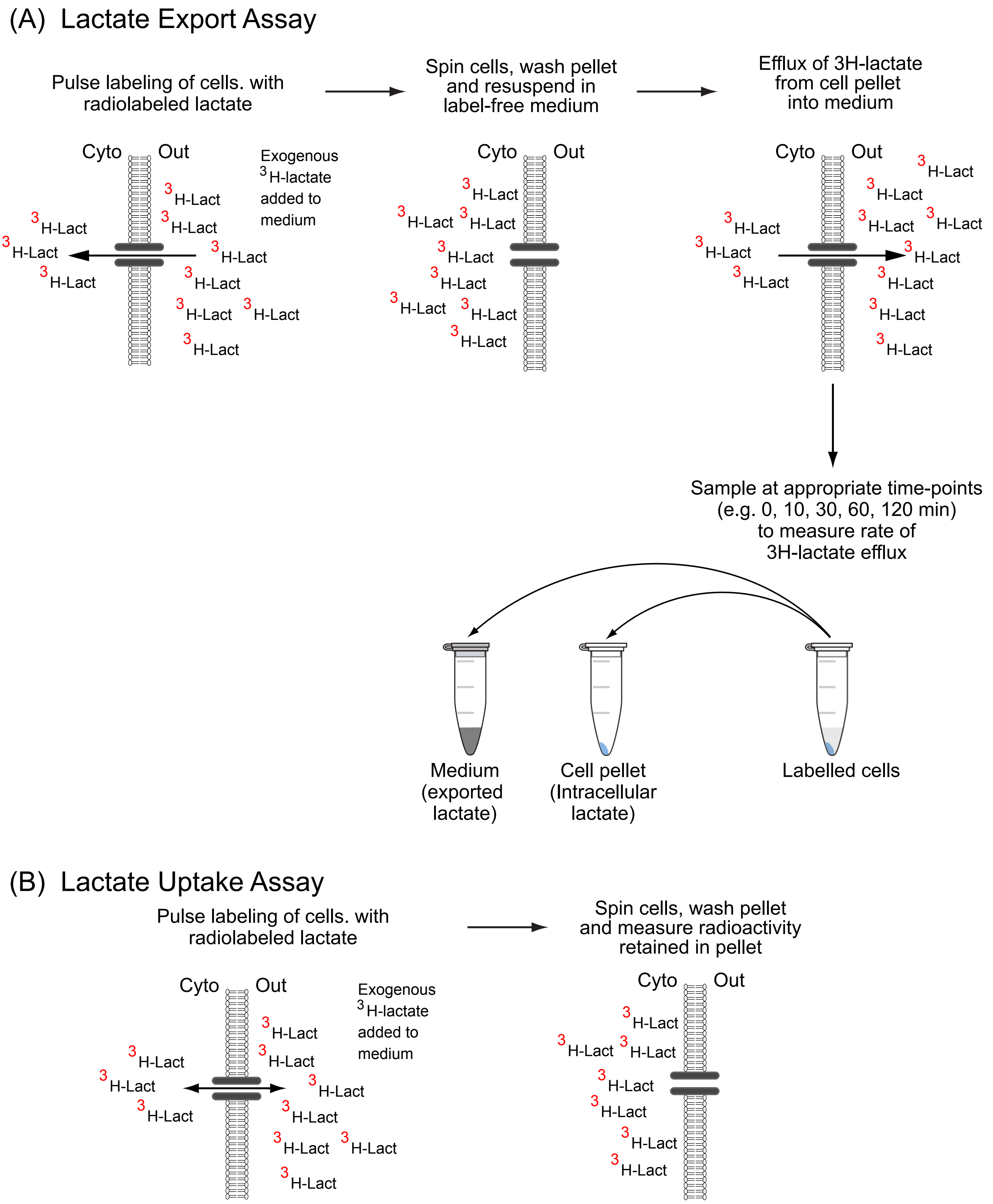

Figure S3. Schematic diagrams of radioactive lactate transport assays. Related to Figures 3 and S4. (A) lactate export assay and **(B)** lactate uptake assay.

Figure S4

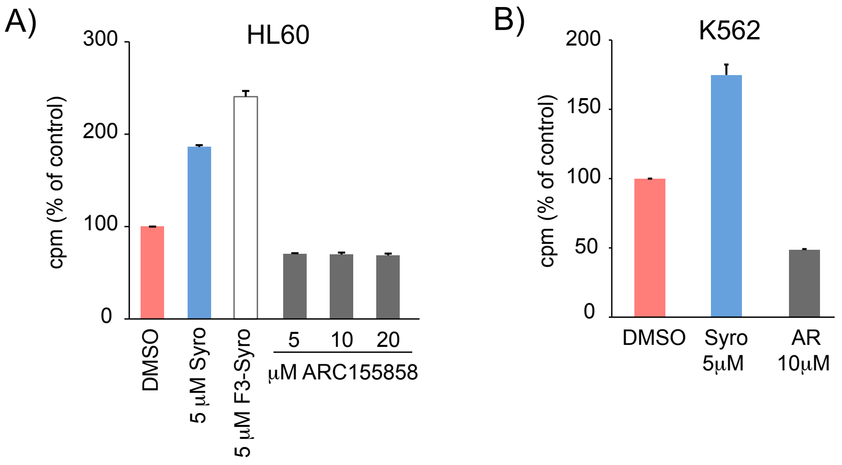

Figure S4. Radioactive lactate uptake and the effect of indicated drugs. Related to Figure 3. Radioactive lactate present in the cell pellet of **(A)** HL60 and **(B)** K562 cells. Labeling pulse was 1 hour. Each data point was measured in duplicate, bars are mean +/- SEM.

Figure S5

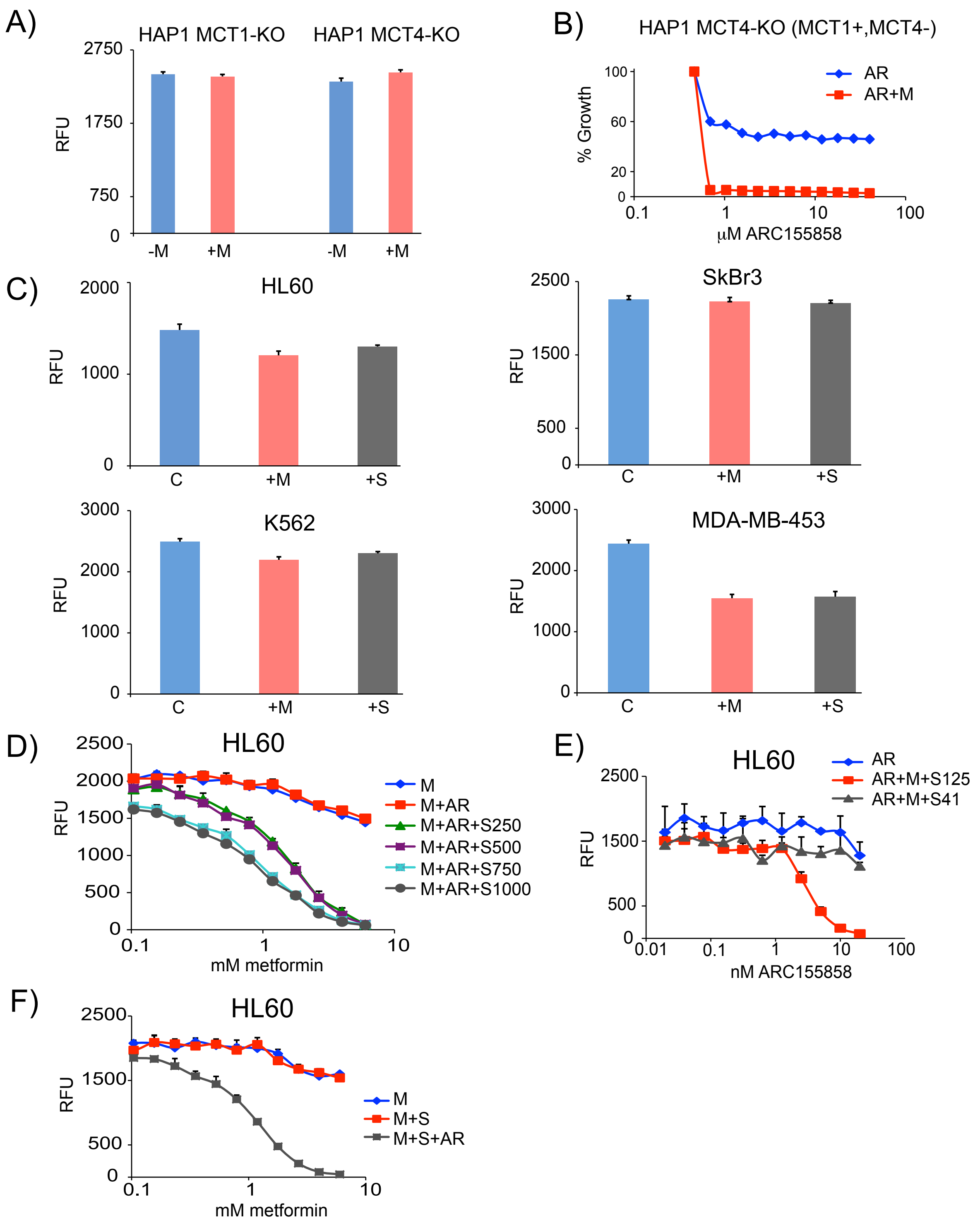

Figure S5. Survival curves of cell line models with varying combinations of metformin, syrosingopine and ARC155858. Related to Figure 5. (A) 4mM metformin (M) has no impact on proliferation in HAP1 MCT1-KO and MCT4-KO cells. **(B)** Survival curve of HAP1 MCT4-KO cells treated with the MCT1 inhibitor ARC155858 (AR), alone or in combination with 4mM metformin. **(C)** Effect of metformin (4mM) and syrosingopine (S, 0.5μM) on proliferation in HL60, K562, SkBr3 and MDA-MB-453 cells. **(D)** Triple drug combination in HL60. Cells were titrated with metformin alone or in a background of 20nM ARC155858 and syrosingopine at various concentrations (250, 500, 750, 1000nM). **(E)** HL60 cells treated with increasing concentrations of ARC155858 in the presence of metformin (4mM) and syrosingopine (41 and 125nM). **(F)** HL60 cells treated with increasing concentrations of metformin in the presence of syrosingopine (1μM) and ARC155858 (20nM). Growth measured after 3 days. Data points were measured in triplicate and show as mean +/- SEM. RFU = relative fluorescence units

Figure S6

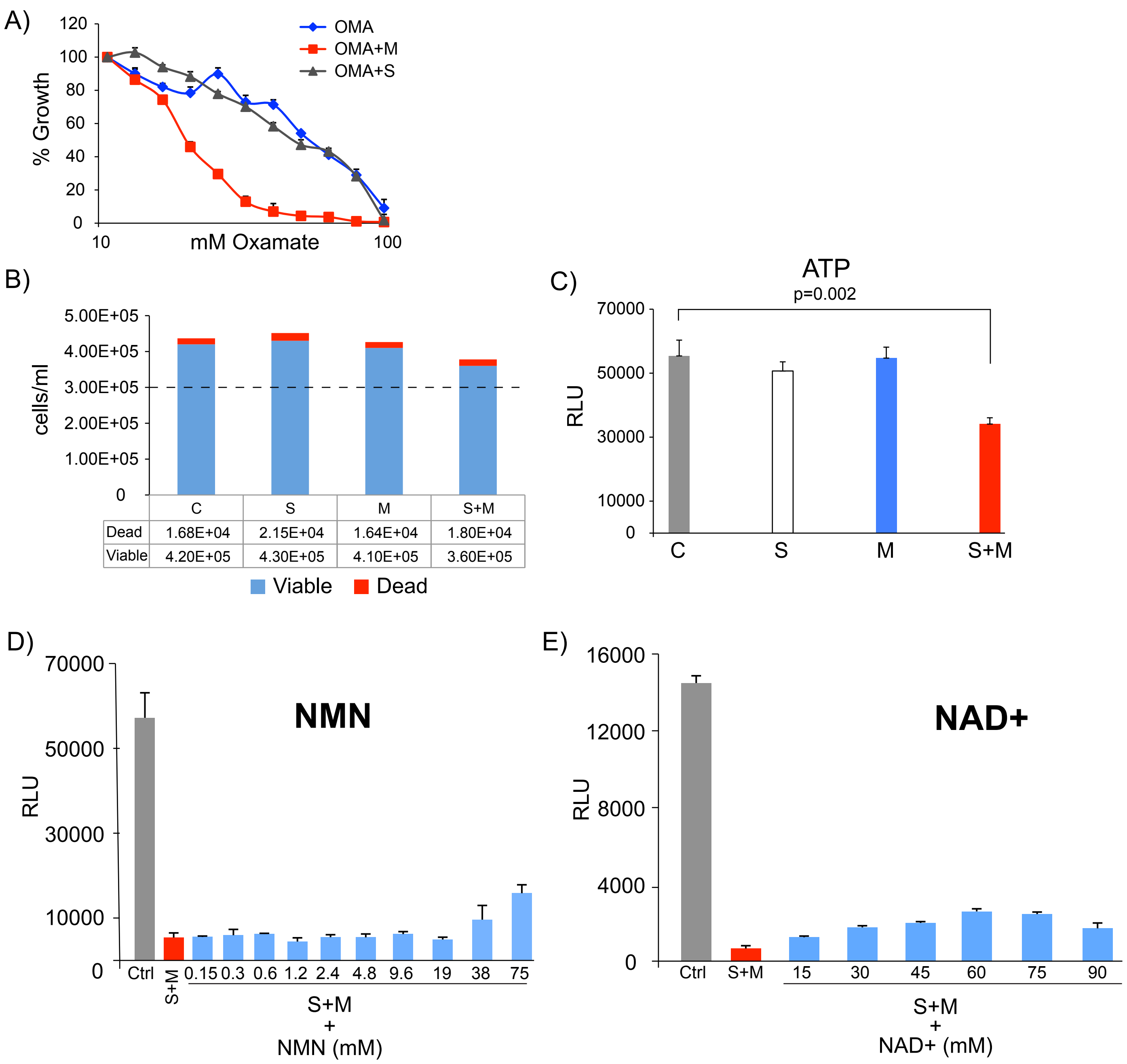

Figure S6. Syrosingopine-metformin treatment impacts NAD+/NADH metabolism. Related to Figure 6. (A) 3-day proliferation assay of HL60 cells titrated with the LDH inhibitor oxamic acid (OMA), and in combination with metformin (M, 4mM) or syrosingopine (S, 4µM). Data points were measured in triplicate, error bars are mean +/- SEM. **(B)** Cell viability counts of HL60 cells after metformin (4mM) or syrosingopine (5µM) treatment for 8 hours, slight growth retardation but no drop in viability was observed for S+M treated cells. Initial cell density shown in dotted line. **(C)** ATP levels of cells in (B) after normalisation for cell numbers. For (B,C) parallel experiments were conducted and data points measured in duplicate. **(D, E)** ATP levels in HL60 cells treated for 30 hours with 5µM syrosingopine and 4mM metformin (S+M). Increasing amounts of NMN or NAD+ were titrated in S+M treated cells. Data points measured in duplicate, bars are mean +/- SEM.
RLU = relative light units
