# Supplementary material for: Dual Inhibition of the Lactate Transporters MCT1 and MCT4 Is Synthetic Lethal with Metformin due to NAD+ Depletion in Cancer Cells
Source: Cell Rep. 2018 Dec 11;25(11):3047–3058.e4. doi: 10.1016/j.celrep.2018.11.043 (PMC6302548; doi:10.1016/j.celrep.2018.11.043)
Supplement: Document S2. Article plus Supplemental Information [file mmc2.pdf]

Cell Reports

Article

Dual Inhibition of the Lactate Transporters MCT1 and MCT4 Is Synthetic Lethal with Metformin due to NAD+ Depletion in Cancer Cells

Graphical Abstract

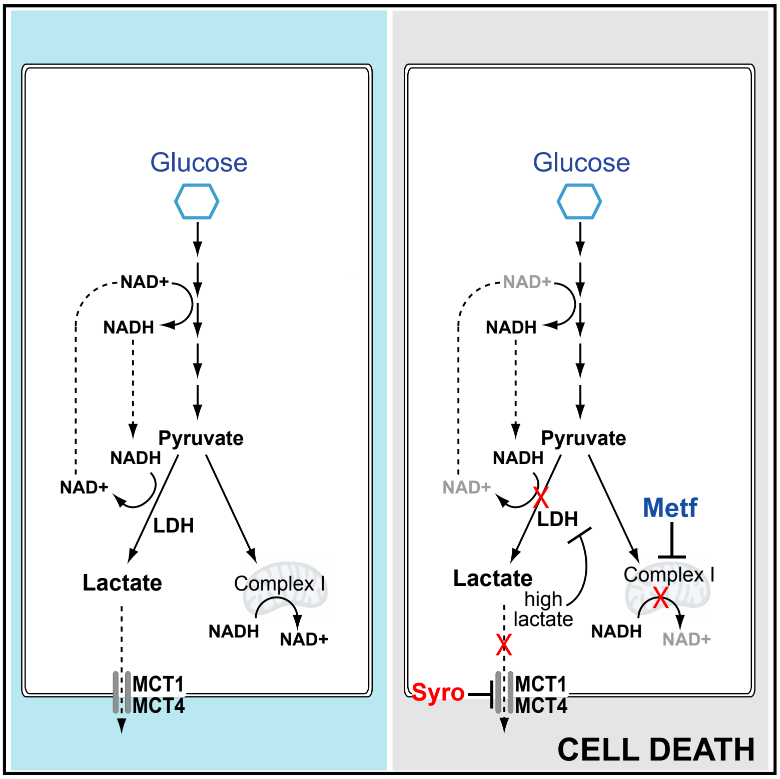

Highlights

- Syrosingopine inhibits lactate export by MCT1 and MCT4
- Combined MCT1 and MCT4 inhibition elicits synthetic lethality with metformin
- Synthetic lethality is due to loss of NAD+-regenerating capacity
- Syrosingopine reduces metformin concentration required for anti-cancer activity

Authors

Don Benjamin, Dimitri Robay, Sravanth K. Hindupur, ..., Christoph Moroni, Heidi A. Lane, Michael N. Hall

Correspondence

heidi.lane@basilea.com (H.A.L.), m.hall@unibas.ch (M.N.H.)

In Brief

Benjamin et al. show that the clinical drug syrosingopine potently inhibits the lactate transporters MCT1 and MCT4 and is thus a clinically relevant MCT4 inhibitor. Intracellular lactate accumulation by syrosingopine elicits synthetic lethality with metformin and potentiates metformin's anti-cancer efficacy.

Benjamin et al., 2018, Cell Reports *25*, 3047–3058
December 11, 2018 © 2018 The Authors.
https://doi.org/10.1016/j.celrep.2018.11.043

CellPress

Cell Reports
Article

OPEN ACCESS
CellPress

Dual Inhibition of the Lactate Transporters MCT1 and MCT4 Is Synthetic Lethal with Metformin due to NAD+ Depletion in Cancer Cells

Don Benjamin,[1,3] Dimitri Robay,[2,3] Sravanth K. Hindupur,[1] Jens Pohlmann,[2] Marco Colombi,[1] Mahmoud Y. El-Shemerly,[2] Sauveur-Michel Maira,[2] Christoph Moroni,[1] Heidi A. Lane,[2,*] and Michael N. Hall[1,4,*]
[1]Biozentrum, University of Basel, 4056 Basel, Switzerland
[2]Basilea Pharmaceutica International Ltd. AG, Basel, Switzerland
[3]These authors contributed equally
[4]Lead Contact
*Correspondence: heidi.lane@basilea.com (H.A.L.), m.hall@unibas.ch (M.N.H.)
https://doi.org/10.1016/j.celrep.2018.11.043

SUMMARY

Highly glycolytic cancer cells prevent intracellular acidification by excreting the glycolytic end-products lactate and H^+ via the monocarboxylate transporters 1 (MCT1) and 4 (MCT4). We report that syrosingopine, an anti-hypertensive drug, is a dual MCT1 and MCT4 inhibitor (with 60-fold higher potency on MCT4) that prevents lactate and H^+ efflux. Syrosingopine elicits synthetic lethality with metformin, an inhibitor of mitochondrial NADH dehydrogenase. NAD+, required for the ATP-generating steps of glycolysis, is regenerated from NADH by mitochondrial NADH dehydrogenase or lactate dehydrogenase. Syrosingopine treatment leads to high intracellular lactate levels and thereby end-product inhibition of lactate dehydrogenase. The loss of NAD+ regeneration capacity due to combined metformin and syrosingopine treatment results in glycolytic blockade, leading to ATP depletion and cell death. Accordingly, ATP levels can be partly restored by exogenously provided NAD+, the NAD precursor nicotinamide mononucleotide (NMN), or vitamin K2. Thus, pharmacological inhibition of MCT1 and MCT4 combined with metformin treatment is a potential cancer therapy.

INTRODUCTION

A hallmark of cancer is a global metabolic shift toward increased glycolysis (Potter et al., 2016). Transformed cells preferentially produce ATP via glycolysis of glucose to lactate. As glycolysis is less efficient than oxidative phosphorylation for ATP generation, cancer cells increase glucose uptake and the glycolytic rate to compensate. This leads to excess lactate production that can cause intracellular acidification. Cytosolic acidification, in turn, reduces the glycolytic rate via inhibition of the rate-limiting enzyme PFK1 (Trivedi and Danforth, 1966). Thus, cells require the efflux of lactate and H^+ to prevent intracellular acidification and to sustain continuously high rates of glycolysis.

The bi-directional monocarboxylate transporters (MCTs) perform H^+-linked transport of L-lactate across the plasma membrane (Halestrap, 2013). Four MCTs are known to transport lactate. MCT1 is ubiquitously expressed and has a high affinity for lactate (3–6 mM). It is the main lactate exporter under normal conditions where intracellular lactate levels are low. Its high affinity for lactate also allows it to import circulating lactate (e.g., in liver where lactate is used for hepatic gluconeogenesis). Some cancers utilize lactate as a metabolic fuel (Faubert et al., 2017; Sonveaux et al., 2008). In solid tumors, zonation of oxygen availability gives rise to tumor symbiosis where lactate excreted by a hypoxic inner core is taken up and used by tumor cells at the vascularized tumor periphery (Allen et al., 2016; Jiménez-Valerio et al., 2016; Pisarsky et al., 2016). In these situations, lactate import by MCT1 supports tumor growth. MCT2 is expressed in brain, liver, and renal tubules. MCT3 is expressed in the choroid plexus and retina. Both MCT2 and MCT3 are poorly studied. MCT4 expression is induced by hypoxia via hypoxia-inducible factor 1 alpha (HIF-1α) (Ullah et al., 2006) and is thus of particular interest in cancer. MCT4 is a marker for poor prognosis in multiple cancers (Baek et al., 2014). MCT4 has a low affinity for lactate (25–30 mM) and does not import serum lactate (normally $<$2mM). Thus, MCT4 appears to be dedicated for lactate export under conditions of high intracellular lactate.

MCT inhibition is a potential therapeutic target in cancer. Pharmacological or genetic ablation of MCT1 or MCT4 activity leads to reduced proliferation *in vitro* and *in vivo* (Le Floch et al., 2011). The anti-proliferative effect of MCT ablation can be augmented with the biguanides metformin and phenformin (Granja et al., 2015; Marchiq et al., 2015). The only effective small-molecule MCT inhibitors developed to date are specific to MCT1, with one drug (AZD3965) currently in clinical trials. However, AZD3965 is ineffective when MCT4 is expressed (Polański et al., 2014), thus restricting its application to tumors that are MCT4−. There has been considerable effort in developing a pan-MCT or MCT4-specific inhibitor due to its potential utility in cancer therapy, but such efforts have not been successful.

This is an open access article under the CC BY license (http://creativecommons.org/licenses/by/4.0/).

We report that syrosingopine inhibits MCT1 and MCT4. We previously described synthetic lethality between syrosingopine and metformin (Benjamin et al., 2016) and now show that this synthetic lethal interaction is due to dual MCT1 and MCT4 inhibition by syrosingopine. Mitochondrial complex I (an NADH dehydrogenase) and lactate dehydrogenase (LDH) are the main cellular sources for regenerating NAD+ required for glycolysis. The direct inhibition of mitochondrial NADH dehydrogenase by metformin, together with the end-product inhibition of LDH due to elevated lactate levels arising from syrosingopine treatment, leads to reduced NAD+ levels. Supplementing NAD+ or increasing endogenous NAD levels with its precursor nicotinamide mononucleotide (NMN) restores ATP levels and delays cell lethality, suggesting that an impaired NAD+ regenerating capacity may be the underlying mechanism of synthetic lethality.

The identification of syrosingopine as a dual MCT1/4 inhibitor can serve as a starting point for further development within this target class. Thus, the rational combination of metformin with syrosingopine, or similar entities with dual MCT1 and MCT4 inhibitory properties, holds promise as an anti-cancer therapy.

RESULTS

Syrosingopine Causes Intracellular Lactate Accumulation and Acidification

Synthetic lethality elicited by the combination of metformin with syrosingopine is accompanied by a decrease in glycolysis, as measured by a drop in ATP and extracellular lactate levels (Benjamin et al., 2016). To further investigate this link, intra- and extra-cellular lactate levels were measured in HeLa cells treated with syrosingopine and various inhibitors of glycolysis and oxidative phosphorylation. We also included reserpine, the parent molecule of syrosingopine, and two novel syrosingopine derivatives F3-syro and SyroD (Figure S1A). Metformin was able to elicit synthetic lethality with the following molecules, in order of decreasing potency: F3-syro, syrosingopine, and reserpine (Figure S1B). Reserpine was previously shown to be less potent than syrosingopine (Benjamin et al., 2016). SyroD, a cytotoxic derivative of syrosingopine, was unable to elicit synthetic lethality with metformin (Figure S1C).

As expected, extracellular lactate levels decreased after glycolysis was inhibited by oxamic acid (OMA) and NaF, which inhibit LDH and enolase, respectively (Figure 1A). Conversely, treatment with inhibitors of oxidative phosphorylation (antimycin A and metformin) increased extracellular lactate levels due to the compensatory upregulation of glycolysis upon inhibition of mitochondrial respiration. Within the syrosingopine compound family, extracellular lactate levels were reduced by syrosingopine and F3-syro, and the magnitude of reduction correlated with the ability to elicit synthetic lethality. Reserpine and SyroD had no effect on extracellular lactate levels.

Intracellular lactate levels were measured in the same samples (Figure 1A). OMA and NaF reduced lactate levels due to the inhibition of glycolysis. The inhibition of oxidative phosphorylation by antimycin A and metformin did not greatly elevate intracellular lactate levels, indicating, in light of the corresponding increase in extracellular lactate, that the additional lactate generated by increased glycolysis is mostly exported out of the cell. Surprisingly, syrosingopine and F3-syro caused a large increase in intracellular lactate levels. No substantial increase was observed with the reserpine or SyroD treatment. Intracellular acidification from lactate accumulation was detectable 3 hr after the addition of syrosingopine and F3-syro (Figure 1B). Accumulation of intracellular lactate by syrosingopine and F3-syro was detectable after 1 hr (Figure 1C) and peaked at 4 hr. The effect was concentration dependent, with F3-syro being more potent than syrosingopine (Figure 1D). Notably, within the compound family, the same hierarchy (F3-syro > syrosingopine > reserpine) was seen for intracellular lactate accumulation and metformin-dependent synthetic lethality, suggesting a possible link.

To determine if intracellular lactate accumulation could be reproduced *in vivo*, we used a liver tumor mouse model (Hindupur et al., 2018), in which syrosingopine-metformin was previously shown to prevent tumor development (Benjamin et al., 2016). Serum lactate levels were significantly reduced in syrosingopine-treated mice (Figure 1E). The intracellular lactate from liver tumor nodules was measured in the same mouse cohort. Higher lactate levels were detected in syrosingopine-treated mice (Figure 1F). Collectively, these data suggest that syrosingopine-treated tumor cells sequester lactate.

Syrosingopine and F3-syro Inhibit the Lactate Transporters MCT1 and MCT4

Accumulation of intracellular lactate was concomitant with reduced extracellular lactate and pointed to a defect in lactate export. Human HAP1 cells were deleted for either MCT1 or MCT4 and target gene knockout was confirmed by immunoblotting (Figure S2A). Clones lacking MCT2 expression were selected. Intracellular lactate levels after treatment with syrosingopine, F3-syro, and a MCT1-specific inhibitor ARC155858 (AR) showed intracellular lactate accumulation in MCT1 knockout (MCT1-KO) cells treated with syrosingopine and F3-syro, indicating that these compounds inhibit MCT4-driven lactate export (Figures 2A and 2D). As expected, the addition of ARC155858 had no effect on lactate transport in the MCT1-KO due to the absence of the drug target. In MCT4-KO cells, ARC155858 induced lactate accumulation via inhibition of MCT1 (Figures 2B and 2E). Syrosingopine and F3-syro treatments also resulted in lactate accumulation in MCT4-KO, indicating that they also inhibit MCT1 in addition to MCT4. Syrosingopine is around 60-fold more potent against MCT4 (half maximal inhibitory concentration [IC_{50}], ~40 nM; Figure 2D) than MCT1 (IC_{50}, ~2500nM; Figure 2E). ARC155858 is highly potent against MCT1 (IC_{50}, ~7 nM; Figure 2E) but has essentially no activity against MCT4 (Figure 2D). Parental HAP1 cells utilize both MCT1 and MCT4 for lactate export. ARC155858 causes lactate accumulation in wild-type cells but to a lower extent than syrosingopine or F3-syro (Figures 2C and 2F), suggesting only partial impairment of lactate transport via MCT1-specific inhibition. These data indicate that syrosingopine and F3-syro inhibit both MCT1 and MCT4, with greater potency against MCT4.

Lactate Efflux by MCT1 and MCT4 Is Inhibited by Syrosingopine and F3-syro

A lactate chase experiment using radioactively labeled lactate was performed to measure lactate export and the effect of

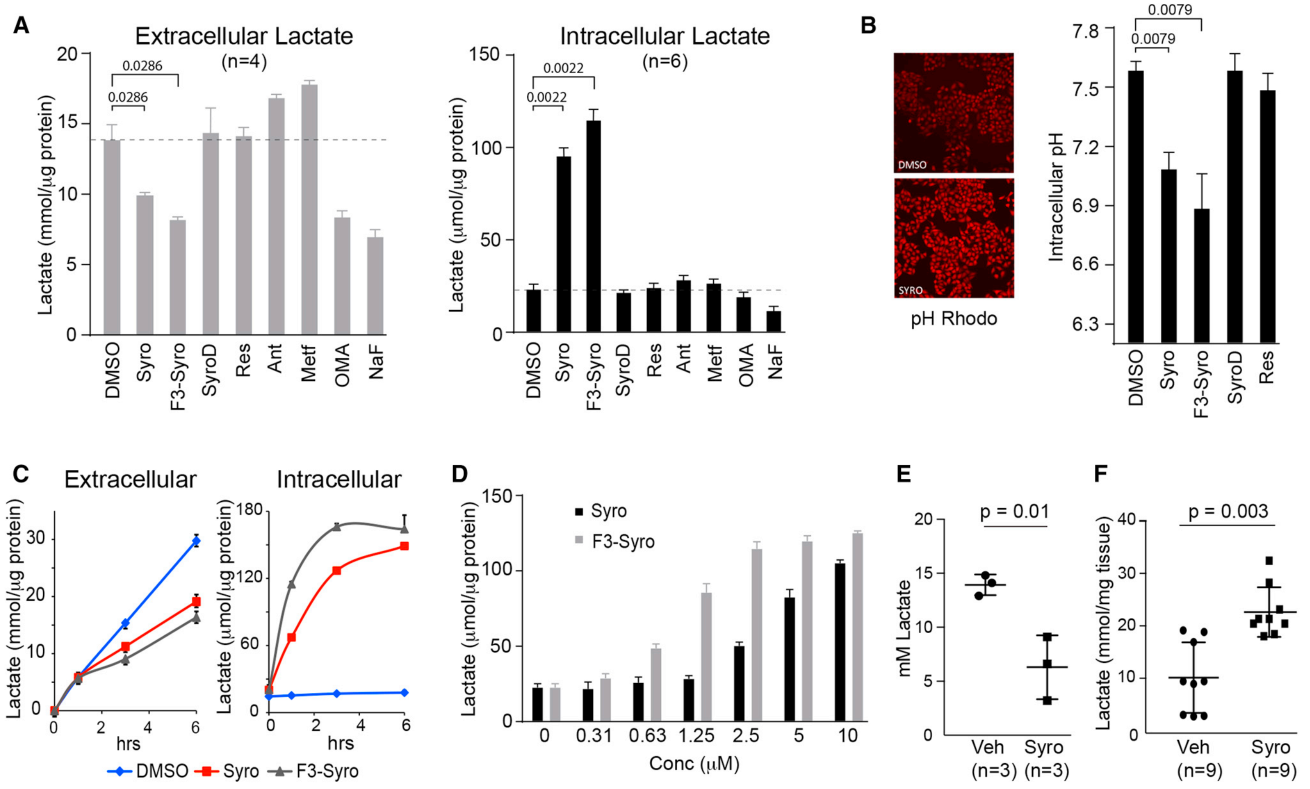

Figure 1. Syrosingopine Causes Intracellular Lactate

(A) HeLa cells were treated for 3 hr with the indicated drugs, and extracellular or intracellular lactate levels were measured (syrosingopine, F3-syro, SyroD, reserpine, 10 μM; antimycin A [Ant], 0.5 μM; metformin, 5mM; oxamic acid [OMA], 20 mM; NaF, 5 mM).

(B) Intracellular pH in drug-treated (10 μM, 3 hr) HeLa cells stained with pHrodo (n = 5).

(C) Rate of extra-or intra-cellular lactate accumulation in HeLa cells treated with indicated drugs (10 μM).

(D) Dose-dependent increase in intracellular lactate levels in response to syrosingopine and F3-syro. HeLa cells were treated for 3 hr.

(E) Serum lactate levels in mice treated with syrosingopine.

(F) Intracellular lactate levels in liver tumor nodules excised from vehicle and syrosingopine treated mice.

Each experiment was performed twice in (A)–(D). Data are presented as mean ± SEM.

drug treatment. Cells were pulse-labeled with ^{3}H-L-lactate, followed by pelleting and re-suspension in label-free medium (Figure S3A). The rate of lactate efflux was determined by measuring the amount of radioactivity retained in the cell pellet or exported to the medium at appropriate time points. Where required, the drug of interest was added during the labeling and post-wash steps.

In MCT4-KO HAP1 cells, lactate efflux from DMSO-treated cells was rapid and essentially complete after 60 min (Figure 3A). Treatment with the MCT1-specific inhibitor ARC155858 blocked lactate efflux as expected, thus validating the assay. Treatment with syrosingopine and F3-syro slowed lactate efflux, with the majority of the label retained in the cell pellet even after 120 min, thus providing direct evidence for MCT1 inhibition by these compounds.

In MCT1-KO cells, treatment with the MCT1-specific inhibitor ARC155858 had no effect on lactate efflux due to the absence of the drug target (Figure 3B). Treatment with syrosingopine and F3-syro slowed lactate efflux, with the majority of the label retained in the cell pellet after 60 min, thus demonstrating that these compounds inhibit MCT4.

HAP1 cells were derived from haploid KBM7 cells that were selected for adherence in tissue culture conditions. To investigate the effect of these drugs in a less manipulated cell background, we screened a panel of well-characterized cancer cell lines for MCT1 and MCT4 expression (Figure S2B). Most cell lines express both transporters, but we identified OPM2 and K562 as being MCT1+ MCT4−, SkBr3 as being MCT1− MCT4+, and MDA-MB-453 as double negative (MCT1− MCT4−). The CD147 chaperone is required for proper folding, translocation, and function of the lactate transporters at the plasma membrane (Kirk et al., 2000) and was present in all the cell lines. We selected HL60 (MCT1+ MCT4+), K562 (MCT1+ MCT4−), SkBr3 (MCT1− MCT4+), and MDA-MB-453 (MCT1− MCT4−) as a cell panel comprising all possible combinations of MCT1 and MCT4 expression. To gauge relative MCT expression levels within the cell panel members, immunodetection of all 4 MCT isoforms was performed on the same blot (Figure S2C). MCT2 was expressed in HL60 and MDA-MB-453, while MCT3 was strongly expressed in MDA-MB-453 and at much lower levels in K562 and SkBr3. Comprehensive determination of the expression profile for all the MCT isoforms thus

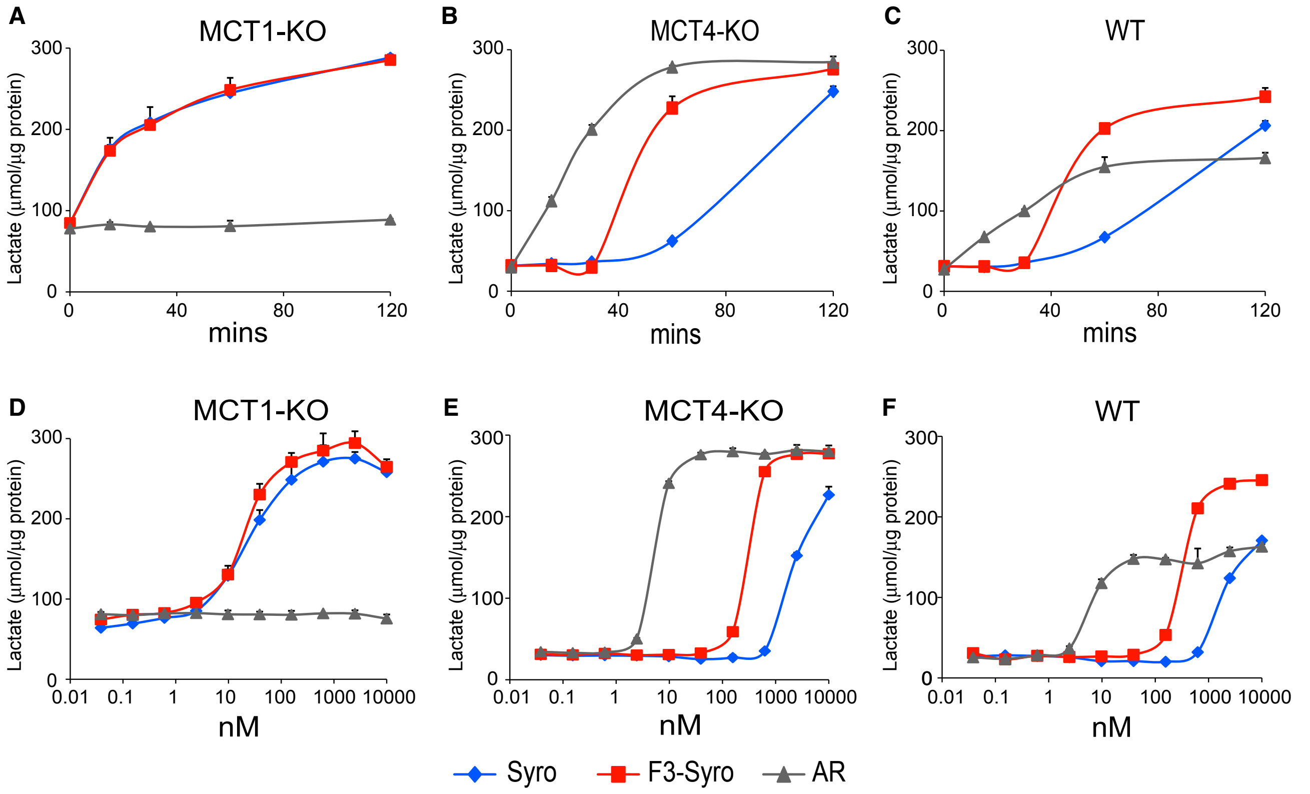

Figure 2. Intracellular Lactate Accumulation Is due to Inhibition of the Lactate Transporters MCT1 and MCT4 by Syrosingopine

(A–C) Intracellular lactate accumulation in HAP1 cells deleted for MCT1 (A), MCT4 (B), and HAP1 wild-type cells (C) following treatment with ARC155858 (1 μM), syrosingopine (10 μM), or F3-syro (10 μM).

(D–F) Dose-responsive increase in intracellular lactate in HAP1 cells deleted for MCT1 (D), MCT4 (E), and HAP1 wild-type cells (F) treated with ARC155858, syrosingopine, and F3-syro for 2 hrs. Each assay was performed twice and a representative experiment is shown. Data points performed in duplicate and presented as mean ± SEM.

revealed that MDA-MB-453 relies on MCT2 and MCT3 for lactate transport, whereas K562 and SkBR3 are mainly reliant on MCT1 and MCT4, respectively. HL60 expressed MCT1, MCT2, and MCT4.

The cell panel was then tested for responsiveness to the combination of metformin with syrosingopine or F3-syro (Figure S1B). Synthetic lethality was elicited in HL60, K562, and SkBr3 but not in MDA-MB-453. Efficacy in HL60 suggests that syrosingopine and F3-syro inhibit MCT1, MCT2, and MCT4. MDA-MB-453 was resistant to the drug combination, indicating that MCT3, which is highly overexpressed only in MDA-MB-453, is not targeted by syrosingopine and F3-syro.

Extra- and intra-cellular lactate levels in response to syrosingopine and F3-syro were measured in HL60 (Figure S2D) and were similar to those previously observed in HeLa cells (Figure 1A). Consequently, we used HL60 to investigate the effect of syrosingopine and F3-syro in the radioactive lactate efflux assay. Syrosingopine and F3-syro strongly retarded lactate efflux, with almost all label retained in the cell at the experimental endpoint (Figure 3C). In contrast, inhibition of MCT1 by ARC155858 did not impede lactate export as MCT2 and MCT4 could compensate for loss of MCT1 activity. Therefore inhibition of lactate transport by syrosingopine and F3-syro in HL60 suggests simultaneous inhibition of MCT1, MCT2, and MCT4 by these drugs.

Lactate Import and Export Are Affected Differently by Syrosingopine and F3-syro

As shown in Figure 3C (0-min time points), lactate uptake was higher in syrosingopine- and F3-syro-treated HL60 cells than in the DMSO control. We repeated labeling of HL60 cells with ^{3}H-L-lactate in the presence of the respective drugs and measured radioactivity in the pellet immediately after the pulse. Lactate transport is bi-directional, and the incorporation at the end of the pulse represents the equilibrium between radiolabel uptake and re-export into the medium (Figure S3B). Lactate uptake was reduced by ARC155858 due to the potent inhibition of MCT1, which is the only isoform that efficiently imports lactate (Figure S4A). However, syrosingopine- and F3-syro-treated cells again displayed increased lactate uptake relative to the DMSO control. Thus, the net effect of the syrosingopine-like drugs acting on both MCT1 and MCT4, after allowing for lactate import and export to reach equilibrium, is to increase the accumulation of labeled lactate in the cell. This seemingly paradoxical accumulation of lactate can be explained if lactate import is less potently inhibited than lactate

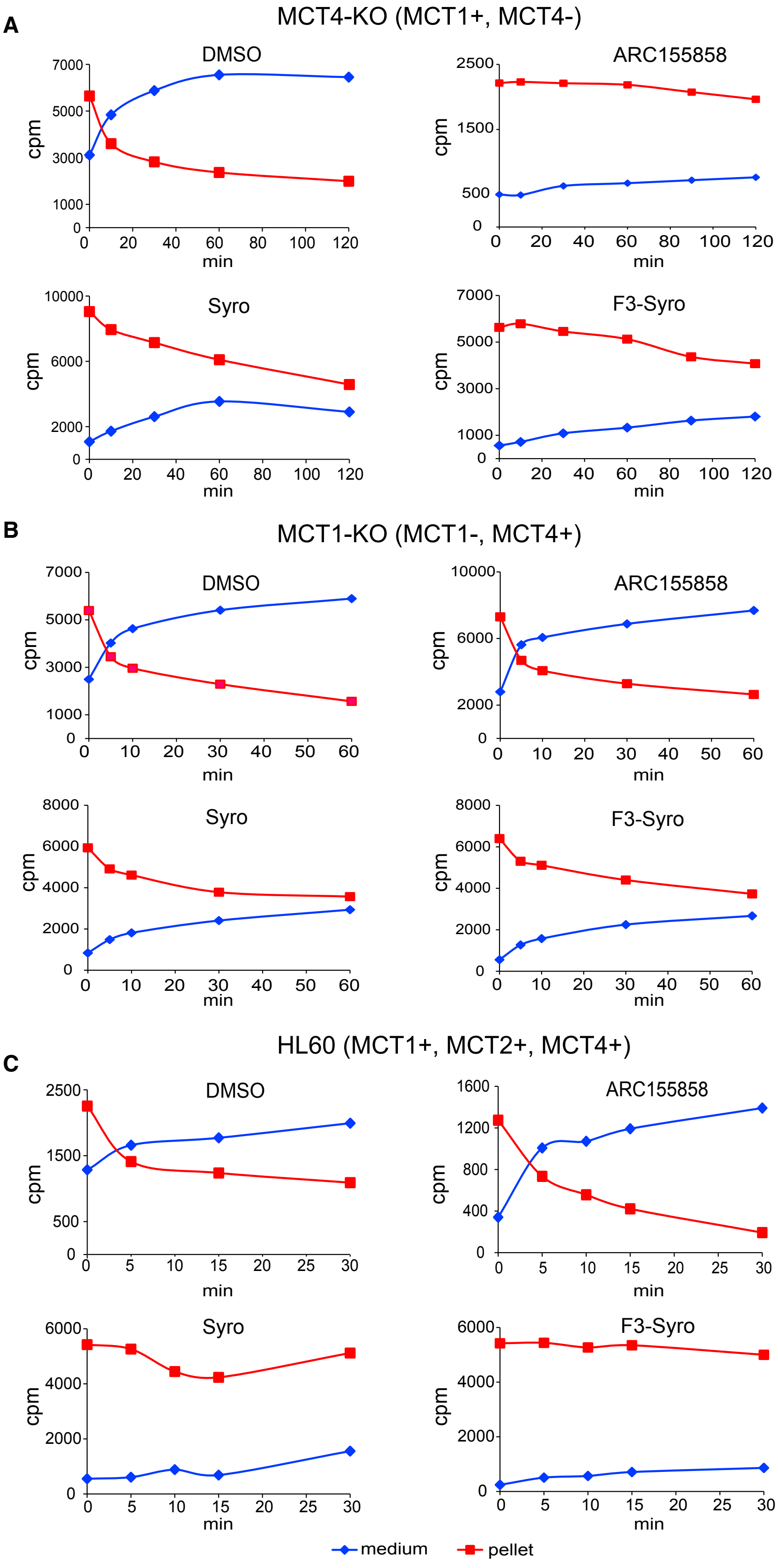

Figure 3. Syrosingopine Inhibits Lactate Efflux by MCT1 and MCT4

Lactate export assays showing amount of radio-labeled lactate (cpm) retained in the cell pellet versus label released into the medium over a time course for HAP1 MCT4-KO cells pre-treated with indicated drugs (syrosingopine, 10 μM; F3-syro, 1 μM; and ARC155858, 10 μM) (A). Similar assays were performed for HAP1 MCT1-KO cells (B) and HL60 cells (C). Each assay was performed at least twice and a representative experiment is shown.

OPEN ACCESS
CellPress

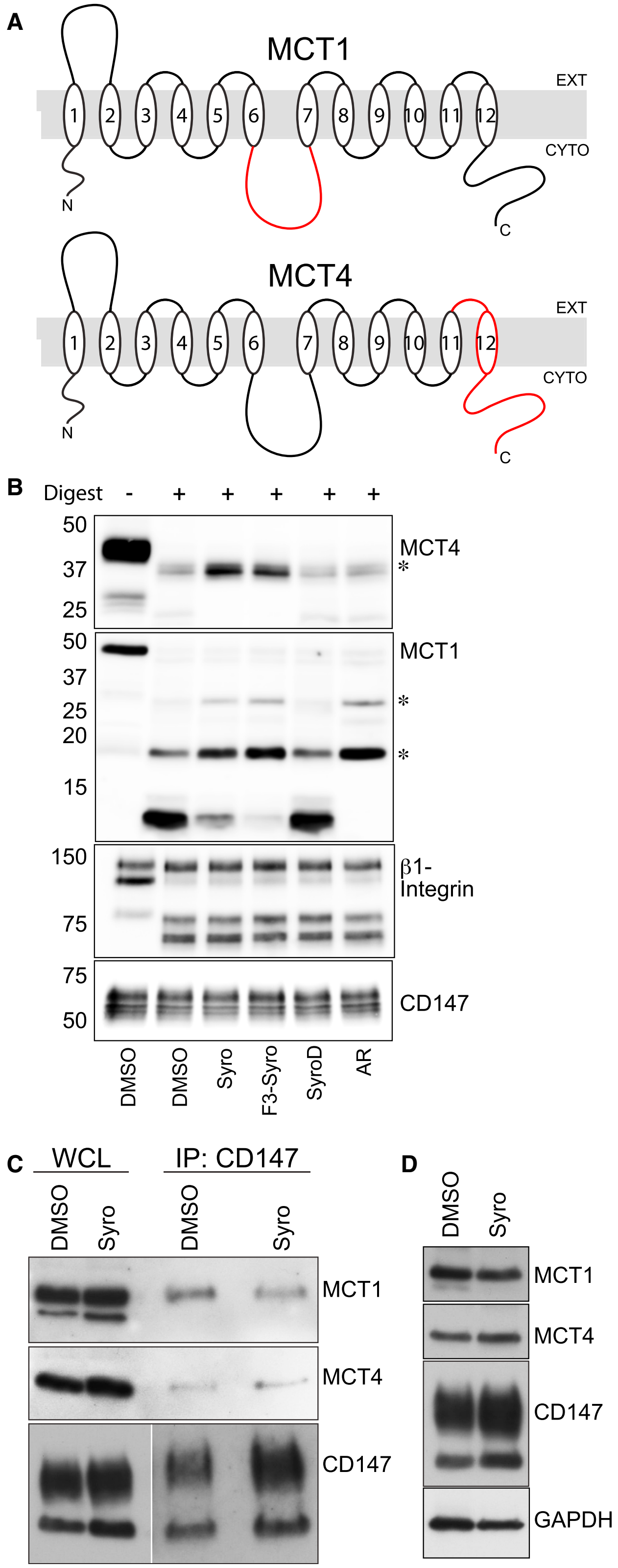

Figure 4. Syrosingopine Interacts Directly with MCT1 and MCT4

(A) Schematic representation of human MCT1 and MCT4 proteins. Epitopes detected by the antibody in red.

(B) DARTS assay from HCT116 cell extracts. Cells were incubated with the indicated compounds (syrosingopine, 10 μM; F3-syro, 10 μM; SyroD, 10 μM; and ARC155858, 10 μM) and lysates subjected to limiting thermolysin digestion. Resulting fragments were probed with antibodies against MCT1, MCT4, CD147, and β1-integrin as control (asterisk indicates protected fragment).

(C) Co-immunoprecipitation from HL60 cells treated with DMSO or syrosingopine (5 μM, 16 hr). The CD147 chaperone was immunoprecipitated and co-IP of MCT1 and MCT4 was determined by immunoblotting (WCL, whole cell lysate).

(D) Levels of CD147, MCT1, and MCT4 proteins in HL60 cells treated for 24 hr with syrosingopine (5 μM).

export, resulting in a net accumulation of exogenous label in the cell.

In K562 cells that only express MCT1, ARC155858 reduced lactate uptake that is consistent with the inhibition of lactate import by MCT1 (Figure S4B). However, net lactate uptake was increased by syrosingopine in K562, indicating that the effect of syrosingopine is greater on lactate export than import in MCT1. These data collectively show that syrosingopine and F3-syro have a different impact on the rate of import and export in the lactate transporters.

Syrosingopine and F3-syro Bind MCT1 and MCT4 *In Vitro*

Drug Affinity Responsive Target Stability (DARTS) was used to investigate possible binding between the syrosingopine class drugs and the MCTs (Lomenick et al., 2009). Human HCT116 colorectal carcinoma cells were incubated with the drug of interest prior to harvest, and membrane extracts were subjected to limiting proteolytic digestion by thermolysin in the presence of the respective drug. Probing for MCT1 resulted in the detection of protected fragments (~18, 28 kDa) in ARC155858-incubated extracts compared to the DMSO control (Figures 4A and 4B) and indicate possible exposed cleavage sites in the intracellular loop (ARC155858 binds MCT1 intracellularly at loops 7–10) (Ovens et al., 2010). Incubation with syrosingopine or F3-syro yielded protected fragments of comparable size as those observed with the ARC155858 treatment (Figure 4B), providing evidence that these compounds interact with MCT1 *in vitro*. The same lysates were probed for MCT4, but there was no evidence for the protection of MCT4 from proteolytic digestion by ARC155858. However, syrosingopine and F3-syro treatment reduced the proteolytic cleavage of a ~37-kDa fragment, suggesting drug binding to MCT4. Notably, the SyroD derivative that is unable to elicit synthetic lethality with metformin also did not confer any protection from proteolytic digestion to MCT1 or MCT4. The MCT4-interacting protein β1-integrin and CD147 either showed non-specific digestion or no digestion at the concentration of thermolysin used and served as internal controls for digestion and protein levels. Overall, these data provide evidence of a direct interaction between syrosingopine and F3-syro with MCT1 and MCT4.

A consequence of drug binding to the lactate transporters may be the disruption of active CD147-MCT complexes. Immunoprecipitation of CD147 showed no change in the association of MCT1 or MCT4 with CD147 upon syrosingopine treatment

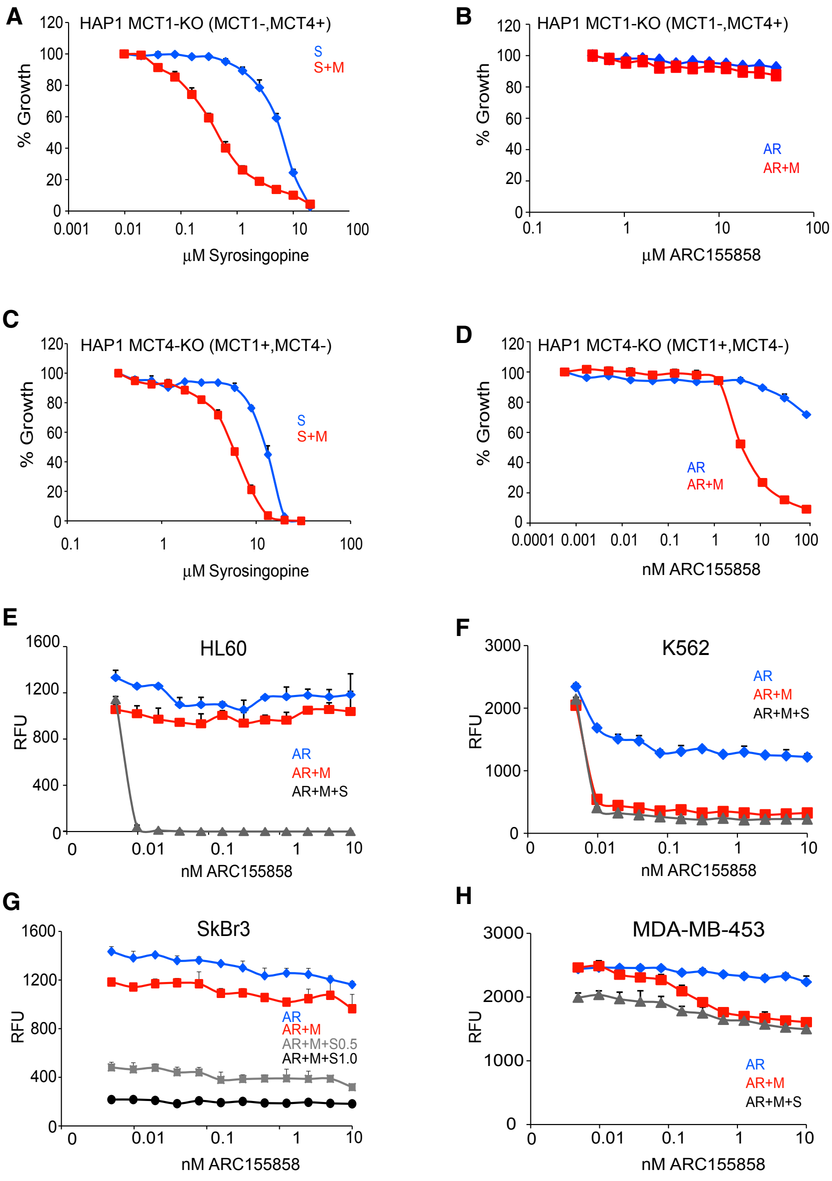

Figure 5. Dual MCT1 and MCT4 Inhibition Is Necessary to Kill Cancer Cells

(A and B) Survival curve of HAP1 MCT1-KO cells with increasing concentrations of syrosingopine (S; in A) and the MCT1 inhibitor ARC155858 (AR; in B). Both drugs were also titrated in the presence of a sub-lethal concentration of metformin (M, 4 mM) to elicit possible synthetic lethality.

(C and D) Survival curve for HAP1 MCT4-KO cells treated with syrosingopine (C) and ARC155858 (D).

(E–H) Human cancer cell lines comprising all combinations of MCT1 and MCT4 expression. HL60 (E), K562 (F), SkBr3 (G), and MDA-MD-453 (H) were titrated with increasing concentrations of ARC155858 in the presence of sub-lethal concentrations of metformin (4 mM) or syrosingopine (0.5 μM). Cell proliferation and survival was measured after 3 days. All data points are in triplicate and presented as mean ± SEM.

(Figure 4C). Prolonged syrosingopine treatment also did not result in a decrease in levels of CD147 or either MCT (Figure 4D). These data support a view that syrosingopine inhibits MCT function directly and not indirectly via an effect on complex formation or stability.

Inhibition of Lactate Transport Is Required for Synthetic Lethality between Syrosingopine and Metformin

Syrosingopine inhibits the vesicular monoamine transporter (Schuldiner et al., 1993); nevertheless, vesicular monoamine transporter (VMAT) inhibition is unrelated to synthetic lethality with metformin (Benjamin et al., 2016). To determine if lactate transport inhibition is responsible for synthetic lethality, HAP1 MCT KO cells were titrated with syrosingopine or ARC155858 in the presence/absence of a non-lethal concentration of metformin (effect of metformin alone is shown in Figure S5A). Synthetic lethality was induced by the syrosingopine-metformin combination in HAP1 MCT1-KO (Figure 5A). As expected, the MCT1-specific inhibitor ARC155858 had no effect regardless of the presence of metformin due to absence of its target (Figure 5B).

Synthetic lethality was induced by syrosingopine-metformin in HAP1 MCT4-KO (Figure 5C). MCT4-KO cells were more sensitive to ARC155858 due to their reliance solely on MCT1 for lactate transport. Nevertheless, MCT1 inhibition, even when it is the only lactate transporter present, was not lethal even at high ARC155858 concentrations but only resulted in reduced cell proliferation (Figure S5B). Instead, MCT1 inhibition by ARC155858 required simultaneous treatment with metformin to elicit lethality (Figures 5D and S5B).

The response to syrosingopine and ARC155858 was studied in further detail by using the cell panel. In HL60, ARC155858 had no effect on growth or survival even in the presence of metformin (Figure 5E), showing that MCT2 and MCT4 are able to compensate for the loss of MCT1 activity. Nonetheless, adding in a low, sub-lethal concentration of syrosingopine to the ARC155858-metformin combination was able to potentiate cell killing, demonstrating that combined MCT1, MCT2, and MCT4 inhibition is absolutely required to elicit cell death. Note that syrosingopine and metformin were used at concentrations where they had a minimal effect on HL60 growth (Figure S5C, and for the other cell lines in the panel). K562 mimicked the results seen in HAP1 MCT4-KO cells. ARC155858 inhibited K562 growth, but lethality was induced only after the addition of metformin (Figure 5F) and increased with the further addition of syrosingopine. Similarly, SkBr3 mimicked the HAP1 MCT1-KO model. SkBr3 proliferation was

unaffected by ARC155858, as expected due to the absence of MCT1, and lethality could not be induced with the addition of metformin (Figure 5G). Increasing the amount of syrosingopine elicited syrosingopine-metformin synthetic lethality, as seen earlier in Figure S1B, but in no case did titrating in higher amounts of ARC155858 result in a dose-dependent increase in cell killing. In MDA-MB-453, ARC155858 had no effect on growth, and the further addition of metformin, or metformin with syrosingopine, was unable to elicit synthetic lethality (Figure 5H).

Collectively, the results from the cell panel, in light of their MCT isoform expression profile (Figure S2C), support the view that the induction of synthetic lethality by syrosingopine with metformin is due to its role as a dual MCT1 and MCT4 inhibitor (while the HL60 results suggest that syrosingopine also inhibits MCT2, there is only direct evidence for MCT1 and MCT4 inhibition from the lactate transport assays). These results (Figures 5A–5H) also demonstrate that the inhibition of lactate transport by itself is not cytotoxic and is lethal only with the concomitant inhibition of oxidative phosphorylation. Furthermore, when MCT4 is expressed, MCT1 inhibition alone is unable to elicit cell death even when oxidative phosphorylation is inhibited by metformin, thus demonstrating the advantage of combined MCT1 and MCT4 inhibition.

The potential of metformin as an anti-cancer therapy may be hampered by inadequate dosing in clinical settings (Chandel et al., 2016; Dowling et al., 2016; Kordes et al., 2015). Metformin was titrated in HL60 against varying concentrations of syrosingopine and ARC155858 to determine the lowest effective concentration of metformin required for cell killing *in vitro* (Figures S5D and S5E). ARC155858 (20 nM) together with syrosingopine (1 μM) was able to reduce the metformin IC_{50} to ~0.9 mM (Figure S5F).

Syrosingopine and Metformin Induce Energy Crisis due to NAD+ Depletion

The accumulation of intracellular lactate does not lead to cell death, as treatment with syrosingopine or ARC155858 is only cytostatic. This result led us to consider the role of NAD+/NADH in cellular energy generation. NAD+ is reduced to NADH during glycolysis, and a constant supply of NAD+ is required to sustain a continuous high rate of glycolysis (Figure 6A). NAD+ is regenerated from NADH via the conversion of pyruvate to lactate by LDH. LDH is end-product inhibited by high lactate concentrations (Stambaugh and Post, 1966). The other major route for NAD+ regeneration is via mitochondrial complex I, the target of metformin. The simultaneous inhibition of LDH and complex I thus results in a loss of the NAD+ regenerating capacity that could lead to NAD+ depletion and glycolytic blockade. LDH inhibition by OMA was shown to be synthetic lethal with phenformin (Miskimins et al., 2014). We observed synthetic lethality between OMA and metformin in HL60 (Figure S6A) but not between OMA and syrosingopine, supporting the idea that OMA and syrosingopine act on the same arm of the synthetic lethal interaction, namely via LDH inhibition.

The NAD+/NADH ratio is a key indicator of cellular health, and a drop in the ratio in response to drug treatment correlates with lower cell proliferation (Gui et al., 2016). Total NAD (NAD+ and NADH) and NADH levels were determined in HL60 treated with syrosingopine, metformin, or in combination. Cells were treated acutely for 8 hr and measurements taken before the onset of cell death arising from syrosingopine-metformin treatment. No significant reduction in cell number or viability was observed by trypan blue staining and automated counting (Figure S6B). Total NAD (NADt) levels were significantly lower after syrosingopine-metformin treatment (Figure 6B). This was due to the loss of NAD+, as reflected by a drop in the NAD+/NADH ratio (Figure 6C) and was concomitant with reduced ATP levels (Figure S6C).

NAD levels can be boosted by supplementation with its precursor NMN (Yoshino et al., 2018). Preliminary experiments showed that syrosingopine-metformin-treated HL60 cells grown in medium supplemented with NMN had a partial recovery in ATP levels (Figure S6D), with similar observations in cells supplemented with NAD+ (Figure S6E). ATP levels were measured in cells treated with syrosingopine-metformin for 30 hr (Figure 6D). High NMN and NAD+ concentrations were needed to counter the effect of syrosingopine-metformin treatment, and this had an impact on growth even in the DMSO controls, but nevertheless, both NMN and NAD+ were able to restore ATP levels after syrosingopine-metformin treatment. HL60 cells start to undergo apoptosis after 24 hr of syrosingopine-metformin treatment (Benjamin et al., 2016) and have a rough, shrunken appearance. However, syrosingopine-metformin-treated cells in the presence of NMN showed less visible signs of damage, indicating a delay in cell death (Figure 6E).

NAD+ levels can be artificially increased by supplementation with quinones, such as vitamin K2 (menaquinone). The reduction of exogenous quinones by cytoplasmic oxidoreductases is coupled with the oxidation of NADH to NAD+ and provides a temporary boost in NAD+ levels before quinone depletion. HL60 cells treated with syrosingopine and metformin show a slight decrease in ATP levels after 8 hr. ATP levels are partially restored in a dose-dependent fashion by exogenous vitamin K2 (Figure 6F).

Collectively, these data support the hypothesis that NAD+ depletion is responsible for synthetic lethality induced by syrosingopine-metformin treatment.

DISCUSSION

We show direct evidence that syrosingopine is a dual inhibitor of the lactate transporters MCT1 and MCT4. Furthermore, we show that dual inhibition of MCT1 and MCT4 accounts for the synthetic lethality of syrosingopine in combination with metformin in human cancer cells.

The MCTs are important for cancer cell growth and survival (Doherty and Cleveland, 2013), and accordingly, a lot of effort has been invested in developing lactate transporter inhibitors as potential anti-cancer agents. Several MCT1-specific inhibitors have been developed (Guile et al., 2006), with one (AZD3965) in phase I clinical trials for advanced cancer. The disadvantage of MCT1-specific inhibition is that it is ineffective when MCT4 is expressed. This is a particularly severe limitation, as MCT4 expression is induced by hypoxia in the majority of tumors. There are no reports of an effective small molecule inhibitor for MCT4. Pouysségur and colleagues have alluded to an

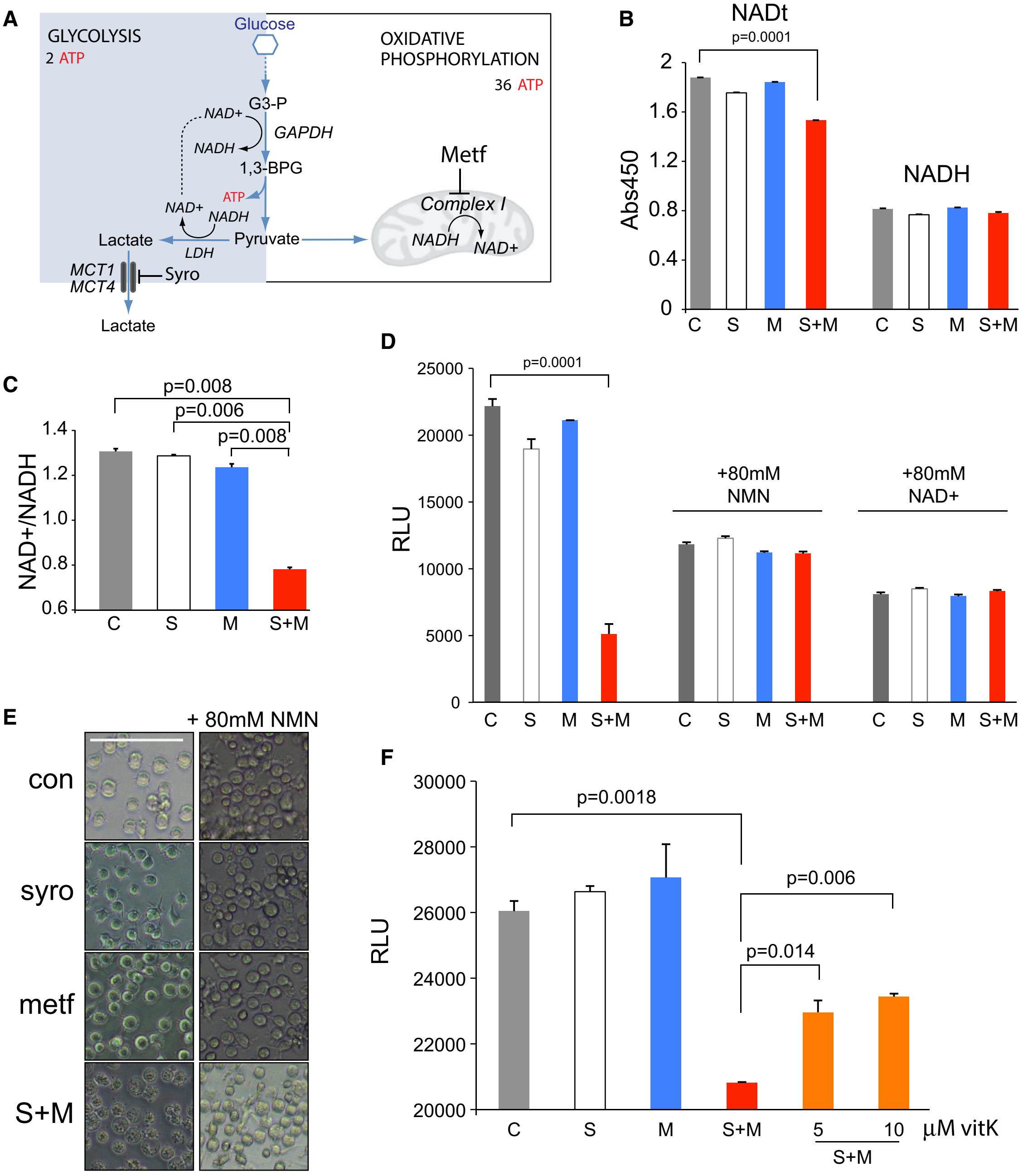

Figure 6. Syrosingopine-Metformin Lethality Can Be Rescued with Exogenous NAD+

(A) Schematic representation of the main branches of the glycolytic pathway.

(B) Total NAD (NADt) and NADH levels in HL60 after metformin (4 mM) or syrosingopine (5 µM) treatment for 8 hr.

(C) NAD+/NADH ratio of HL60 cells in (B).

(D) ATP levels measured in HL60 cells after 30 hr of treatment with metformin (4 mM) or syrosingopine (5 µM) and parallel treatment in the presence of NMN or NAD+.

(E) Micrographs of HL60 cells treated as in (D). Scale bar represents 100 µm.

(F) ATP levels in HL60 cells treated with metformin (4 mM) or syrosingopine (5 µM) for 8 hr and with the addition of vitamin K2. All experiments were performed twice. Data points were performed in duplicate and presented as mean ± SEM. RLU, relative light units.

MCT4-specific inhibitor, AZ93 (Marchiq and Pouysségur, 2016). However, similar to MCT1-specific inhibitors, this compound is ineffective in cells expressing both MCT1 and MCT4 due to the functional redundancy of the transporters. Acriflavine was recently reported to disrupt the MCT4-CD147 interaction (but not MCT1-CD147), but this had no effect on lactate secretion

(Voss et al., 2017). We see no evidence that syrosingopine disrupts the interaction between MCT1 or MCT4 and CD147; instead, syrosingopine appears to interact directly with the transporters (Figure 4). Diclofenac was shown to prevent lactate uptake by MCT4 in a *Xenopus* oocyte lactate transport assay (Sasaki et al., 2016); however, its inhibitory effect on lactate uptake in human Caco-2 cells is unclear, as MCT isoform expression was not characterized. We show direct biochemical evidence that syrosingopine inhibits lactate transport by MCT1 and MCT4. In addition, synthetic lethality between syrosingopine and metformin in a cell panel comprising various combinations of MCT1-4 isoform expression suggests that syrosingopine is also able to inhibit MCT2 but is inactive against MCT3.

How does syrosingopine in combination with metformin elicit synthetic lethality? Under physiological conditions, the reduction of pyruvate to lactate by LDH is favored and serves to regenerate NAD+ consumed upstream in the ATP-producing steps of the glycolytic pathway (Figure 6A). Lactate accumulation upon MCT inhibition leads to high intracellular lactate concentrations that can result in end-product inhibition of LDH and, consequently, loss of NAD+ regenerating capacity. The simultaneous inhibition by metformin of mitochondrial complex I, the other main source of NAD+ regeneration, results in a decrease in the NAD+/NADH ratio, loss of glycolytic ATP production, and cell death. The observation that ATP levels can be partly restored by exogenous NAD+ or the NAD precursor NMN suggests that the cause of synthetic lethality is NAD+ depletion. This rescue requires supra-physiological concentrations of NAD+ and NMN, which may be due to the poor permeability of these compounds in HL60 cells (Billington et al., 2008). We note that NAD+ and NMN are unable to prevent cell death after 48 hr of syrosingopine-metformin treatment (data not shown). The lack of NAD+ to fuel glycolysis is a plausible reason for syrosingopine-metformin synthetic lethality. This is reminiscent of the situation in DNA-damaged cells where activated PARP1 consumes excessive amounts of NAD+ (Ying et al., 2005), leading to cell death. Vitamin K2 partially rescues ATP production in syrosingopine-metformin-treated cells. Exogenous vitamin K2 gives a temporary boost in NAD+ levels that transiently supports glycolysis despite syrosingopine-metformin treatment. The rescue is short-lived due to the depletion of exogenous vitamin K2 and the absence of NAD+/NADH-regenerating mechanisms, which unfortunately precludes the opportunity of studying an effect on cell proliferation. Nevertheless, it provides supporting evidence for the proposed mechanism of synthetic lethality.

Syrosingopine, as a dual MCT1 and MCT4 inhibitor, may have additional anti-tumor benefits *in vivo*. Most of the lactate secreted by cancer cells accumulates in the extracellular space, creating a tumor microenvironment that promotes cell invasion and metastasis (Kato et al., 2013). Extracellular acidification by lactate also has an immunosuppressive effect on tumor-infiltrating immune cells (Brand et al., 2016; Fischer et al., 2007). Functional differentiation in some tumors into highly glycolytic hypoxic cores surrounded by well-vascularized outer regions results in metabolic symbiosis where lactate generated as a waste product in the hypoxic core is utilized as a fuel by normoxic cancer cells at the tumor periphery. Tumor cells can also utilize lactate originating from surrounding stromal cells (the reverse Warburg effect) or directly take up lactate from the circulation (Faubert et al., 2017; Pavlides et al., 2009). Thus ,in all these scenarios, syrosingopine-mediated trapping of lactate in tumor cells can provide an additional bonus beyond the effect of the drug combination on glycolysis.

There is great interest in re-positioning metformin as an anti-cancer drug, and numerous clinical trials have been initiated to assess its anti-cancer activity. Concluded trials have reported mixed results, showing either no or weak clinical efficacy (Kordes et al., 2015; Tsilidis et al., 2014). There is considerable debate on the effective metformin concentration required for anti-neoplastic activity (Chandel et al., 2016; Dowling et al., 2016). The metformin concentration used in pre-clinical models demonstrating anti-cancer activity (mM range) is an order of magnitude greater than the serum metformin concentration attainable with routine anti-diabetic dosing (μM range), suggesting that this may be partly the reason behind the mixed results from the clinical trials. In this light, the ability of syrosingopine to elicit synthetic lethality with metformin in cancer cells and to substantially lower the effective concentration of metformin required in cellular models (Figure S5F) may be of potential clinical benefit. As lactate transport inhibition alone is at best cytostatic, this suggests that the rational combination of an MCT1 and MCT4 inhibitor with metformin may prove a viable anti-cancer strategy for both drug classes.

STAR★METHODS

Detailed methods are provided in the online version of this paper and include the following:

- KEY RESOURCES TABLE
- CONTACT FOR REAGENT AND RESOURCE SHARING
- EXPERIMENTAL MODEL AND SUBJECT DETAILS
  - Mouse Liver tumor model
- METHOD DETAILS
  - Cell proliferation assays
  - Immunoblotting
  - Co-immunoprecipitation
  - DARTS assay
  - Mouse experiments
  - ATP and lactate measurement
  - Intracellular pH measurement
  - Radiolabeled lactate uptake and export assays
  - NAD+/NADH measurements
- QUANTIFICATION AND STATISTICAL ANALYSIS

SUPPLEMENTAL INFORMATION

Supplemental Information includes six figures and can be found with this article online at https://doi.org/10.1016/j.celrep.2018.11.043.

ACKNOWLEDGMENTS

This work was supported by the Swiss National Science Foundation, the Louis-Jeantet Foundation, the European Research Council (MERiC), and the Canton of Basel.

AUTHOR CONTRIBUTIONS

Conceptualization, D.B., D.R., H.A.L., and M.N.H.; Investigation, D.B., D.R., S.K.H., and M.C.; Writing – Original Draft, D.B. and D.R.; Writing – Review & Editing, H.A.L., C.M., and M.N.H.; Funding Acquisition, H.A.L. and M.N.H.; Resources, J.P., M.Y.E.-S., and S.M.M.

DECLARATION OF INTEREST

The authors declare no competing interests.

Received: June 22, 2018
Revised: October 17, 2018
Accepted: November 9, 2018
Published: December 11, 2018

REFERENCES

Allen, E., Miéville, P., Warren, C.M., Saghafinia, S., Li, L., Peng, M.-W., and Hanahan, D. (2016). Metabolic symbiosis enables adaptive resistance to anti-angiogenic therapy that is dependent on mTOR signaling. Cell Rep. *15*, 1144–1160.

Baek, G., Tse, Y.F., Hu, Z., Cox, D., Buboltz, N., McCue, P., Yeo, C.J., White, M.A., DeBerardinis, R.J., Knudsen, E.S., and Witkiewicz, A.K. (2014). MCT4 defines a glycolytic subtype of pancreatic cancer with poor prognosis and unique metabolic dependencies. Cell Rep. *9*, 2233–2249.

Benjamin, D., Colombi, M., Hindupur, S.K., Betz, C., Lane, H.A., El-Shemerly, M.Y.M., Lu, M., Quagliata, L., Terracciano, L., Moes, S., et al. (2016). Syrosingopine sensitizes cancer cells to killing by metformin. Sci. Adv. *2*, e1601756.

Billington, R.A., Travelli, C., Ercolano, E., Galli, U., Roman, C.B., Grolla, A.A., Canonico, P.L., Condorelli, F., and Genazzani, A.A. (2008). Characterization of NAD uptake in mammalian cells. J. Biol. Chem. *283*, 6367–6374.

Brand, A., Singer, K., Koehl, G.E., Kolitzus, M., Schoenhammer, G., Thiel, A., Matos, C., Bruss, C., Klobuch, S., Peter, K., et al. (2016). LDHA-associated lactic acid production blunts tumor immunosurveillance by T and NK cells. Cell Metab. *24*, 657–671.

Chandel, N.S., Avizonis, D., Reczek, C.R., Weinberg, S.E., Menz, S., Neuhaus, R., Christian, S., Haegebarth, A., Algire, C., and Pollak, M. (2016). Are metformin doses used in murine cancer models clinically relevant? Cell Metab. *23*, 569–570.

Doherty, J.R., and Cleveland, J.L. (2013). Targeting lactate metabolism for cancer therapeutics. J. Clin. Invest. *123*, 3685–3692.

Dowling, R.J.O., Lam, S., Bassi, C., Mouaaz, S., Aman, A., Kiyota, T., Al-Awar, R., Goodwin, P.J., and Stambolic, V. (2016). Metformin pharmacokinetics in mouse tumors: implications for human therapy. Cell Metab. *23*, 567–568.

Faubert, B., Li, K.Y., Cai, L., Hensley, C.T., Kim, J., Zacharias, L.G., Yang, C., Do, Q.N., Doucette, S., Burguete, D., et al. (2017). Lactate metabolism in human lung tumors. Cell *171*, 358–371.e9.

Fischer, K., Hoffmann, P., Voelkl, S., Meidenbauer, N., Ammer, J., Edinger, M., Gottfried, E., Schwarz, S., Rothe, G., Hoves, S., et al. (2007). Inhibitory effect of tumor cell-derived lactic acid on human T cells. Blood *109*, 3812–3819.

Granja, S., Marchiq, I., Le Floch, R., Moura, C.S., Baltazar, F., and Pouysségur, J. (2015). Disruption of BASIGIN decreases lactic acid export and sensitizes non-small cell lung cancer to biguanides independently of the LKB1 status. Oncotarget *6*, 6708–6721.

Gui, D.Y., Sullivan, L.B., Luengo, A., Hosios, A.M., Bush, L.N., Gitego, N., Davidson, S.M., Freinkman, E., Thomas, C.J., and Vander Heiden, M.G. (2016). Environment dictates dependence on mitochondrial complex I for NAD+ and aspartate production and determines cancer cell sensitivity to metformin. Cell Metab. *24*, 716–727.

Guile, S.D., Bantick, J.R., Cheshire, D.R., Cooper, M.E., Davis, A.M., Donald, D.K., Evans, R., Eyssade, C., Ferguson, D.D., Hill, S., et al. (2006). Potent blockers of the monocarboxylate transporter MCT1: novel immunomodulatory compounds. Bioorg. Med. Chem. Lett. *16*, 2260–2265.

Halestrap, A.P. (2013). The SLC16 gene family - structure, role and regulation in health and disease. Mol. Aspects Med. *34*, 337–349.

Hindupur, S.K., Colombi, M., Fuhs, S.R., Matter, M.S., Guri, Y., Adam, K., Cornu, M., Piscuoglio, S., Ng, C.K.Y., Betz, C., et al. (2018). The protein histidine phosphatase LHPP is a tumour suppressor. Nature *555*, 678–682.

Jiménez-Valerio, G., Martínez-Lozano, M., Bassani, N., Vidal, A., Ochoa-de-Olza, M., Suárez, C., García-Del-Muro, X., Carles, J., Viñals, F., Graupera, M., et al. (2016). Resistance to antiangiogenic therapies by metabolic symbiosis in renal cell carcinoma PDX models and patients. Cell Rep. *15*, 1134–1143.

Kato, Y., Ozawa, S., Miyamoto, C., Maehata, Y., Suzuki, A., Maeda, T., and Baba, Y. (2013). Acidic extracellular microenvironment and cancer. Cancer Cell Int. *13*, 89.

Kirk, P., Wilson, M.C., Heddle, C., Brown, M.H., Barclay, A.N., and Halestrap, A.P. (2000). CD147 is tightly associated with lactate transporters MCT1 and MCT4 and facilitates their cell surface expression. EMBO J. *19*, 3896–3904.

Kordes, S., Pollak, M.N., Zwinderman, A.H., Mathôt, R.A., Weterman, M.J., Beeker, A., Punt, C.J., Richel, D.J., and Wilmink, J.W. (2015). Metformin in patients with advanced pancreatic cancer: a double-blind, randomised, placebo-controlled phase 2 trial. Lancet Oncol. *16*, 839–847.

Le Floch, R., Chiche, J., Marchiq, I., Naiken, T., Ilc, K., Murray, C.M., Critchlow, S.E., Roux, D., Simon, M.P., and Pouysségur, J. (2011). CD147 subunit of lactate/H+ symporters MCT1 and hypoxia-inducible MCT4 is critical for energetics and growth of glycolytic tumors. Proc. Natl. Acad. Sci. USA *108*, 16663–16668.

Lomenick, B., Hao, R., Jonai, N., Chin, R.M., Aghajan, M., Warburton, S., Wang, J., Wu, R.P., Gomez, F., Loo, J.A., et al. (2009). Target identification using drug affinity responsive target stability (DARTS). Proc. Natl. Acad. Sci. USA *106*, 21984–21989.

Marchiq, I., and Pouysségur, J. (2016). Hypoxia, cancer metabolism and the therapeutic benefit of targeting lactate/H(+) symporters. J. Mol. Med. (Berl.) *94*, 155–171.

Marchiq, I., Le Floch, R., Roux, D., Simon, M.-P., and Pouysségur, J. (2015). Genetic disruption of lactate/H+ symporters (MCTs) and their subunit CD147/BASIGIN sensitizes glycolytic tumor cells to phenformin. Cancer Res. *75*, 171–180.

Miskimins, W.K., Ahn, H.J., Kim, J.Y., Ryu, S., Jung, Y.-S., and Choi, J.Y. (2014). Synergistic anti-cancer effect of phenformin and oxamate. PLoS ONE *9*, e85576.

Ovens, M.J., Davies, A.J., Wilson, M.C., Murray, C.M., and Halestrap, A.P. (2010). AR-C155858 is a potent inhibitor of monocarboxylate transporters MCT1 and MCT2 that binds to an intracellular site involving transmembrane helices 7-10. Biochem. J. *425*, 523–530.

Pavlides, S., Whitaker-Menezes, D., Castello-Cros, R., Flomenberg, N., Witkiewicz, A.K., Frank, P.G., Casimiro, M.C., Wang, C., Fortina, P., Addya, S., et al. (2009). The reverse Warburg effect: aerobic glycolysis in cancer associated fibroblasts and the tumor stroma. Cell Cycle *8*, 3984–4001.

Pisarsky, L., Bill, R., Fagiani, E., Dimeloe, S., Goosen, R.W., Hagmann, J., Hess, C., and Christofori, G. (2016). Targeting metabolic symbiosis to overcome resistance to anti-angiogenic therapy. Cell Rep. *15*, 1161–1174.

Polański, R., Hodgkinson, C.L., Fusi, A., Nonaka, D., Priest, L., Kelly, P., Trapani, F., Bishop, P.W., White, A., Critchlow, S.E., et al. (2014). Activity of the monocarboxylate transporter 1 inhibitor AZD3965 in small cell lung cancer. Clin. Cancer Res. *20*, 926–937.

Potter, M., Newport, E., and Morten, K.J. (2016). The Warburg effect: 80 years on. Biochem. Soc. Trans. *44*, 1499–1505.

Sasaki, S., Futagi, Y., Ideno, M., Kobayashi, M., Narumi, K., Furugen, A., and Iseki, K. (2016). Effect of diclofenac on SLC16A3/MCT4 by the Caco-2 cell line. Drug Metab. Pharmacokinet. *31*, 218–223.

Schuldiner, S., Liu, Y., and Edwards, R.H. (1993). Reserpine binding to a vesicular amine transporter expressed in Chinese hamster ovary fibroblasts. J. Biol. Chem. *268*, 29–34.

Sonveaux, P., Végran, F., Schroeder, T., Wergin, M.C., Verrax, J., Rabbani, Z.N., De Saedeleer, C.J., Kennedy, K.M., Diepart, C., Jordan, B.F., et al.

(2008). Targeting lactate-fueled respiration selectively kills hypoxic tumor cells in mice. J. Clin. Invest. *118*, 3930–3942.

Stambaugh, R., and Post, D. (1966). Substrate and product inhibition of rabbit muscle lactic dehydrogenase heart (H4) and muscle (M4) isozymes. J. Biol. Chem. *241*, 1462–1467.

Trivedi, B., and Danforth, W.H. (1966). Effect of pH on the kinetics of frog muscle phosphofructokinase. J. Biol. Chem. *241*, 4110–4112.

Tsilidis, K.K., Capothanassi, D., Allen, N.E., Rizos, E.C., Lopez, D.S., van Veldhoven, K., Sacerdote, C., Ashby, D., Vineis, P., Tzoulaki, I., and Ioannidis, J.P.A. (2014). Metformin does not affect cancer risk: a cohort study in the U.K. Clinical Practice Research Datalink analyzed like an intention-to-treat trial. Diabetes Care *37*, 2522–2532.

Ullah, M.S., Davies, A.J., and Halestrap, A.P. (2006). The plasma membrane lactate transporter MCT4, but not MCT1, is up-regulated by hypoxia through a HIF-1alpha-dependent mechanism. J. Biol. Chem. *281*, 9030–9037.

Voss, D.M., Spina, R., Carter, D.L., Lim, K.S., Jeffery, C.J., and Bar, E.E. (2017). Disruption of the monocarboxylate transporter-4-basigin interaction inhibits the hypoxic response, proliferation, and tumor progression. Sci. Rep. *7*, 4292.

Ying, W., Alano, C.C., Garnier, P., and Swanson, R.A. (2005). NAD+ as a metabolic link between DNA damage and cell death. J. Neurosci. Res. *79*, 216–223.

Yoshino, J., Baur, J.A., and Imai, S.-I. (2018). NAD^+ intermediates: the biology and therapeutic potential of NMN and NR. Cell Metab. *27*, 513–528.

STAR★METHODS

KEY RESOURCES TABLE

REAGENT or RESOURCE	SOURCE	IDENTIFIER
Antibodies		
Rabbit polyclonal MCT1	Santa Cruz	sc50324; RRID: AB_2189197
Mouse monoclonal MCT1	Santa Cruz	sc365501; RRID: AB_10841766
Rabbit polyclonal MCT2	Santa Cruz	sc50322; RRID: AB_2187242
Rabbit polyclonal MCT3	Abcam	ab60333; RRID AB_944129
Rabbit polyclonal MCT4	Santa Cruz	sc50329; RRID AB_2189333
Mouse monoclonal MCT4	Santa Cruz	sc376140; RRID AB_10992036
Mouse monoclonal CD147	R&D	MAB972; RRID AB_2066679
Rabbit polyclonal CD147	Abcam	ab64616; RRID AB_1603445
Rabbit polyclonal GAPDH	Abcam	ab9385; RRID AB_449791
Mouse monoclonal β1-integrin	Santa Cruz	sc374430; RRID AB_10991321
Chemicals, Peptides, and Recombinant Proteins		
Syrosingopine	Extrasynthese	#1691; CAS 84-36-6
F3-Syro	Custom synthesis	N/A
Syro-D	Custom synthesis	N/A
Reserpine	Sigma-Aldrich	R0875; CAS 50-55-5
ARC155858	Tocris	CAS 496791-37-8
Sodium oxamate	Sigma-Aldrich	O2751; CAS 565-73-1
Vitamin K2	Sigma-Aldrich	V9378; CAS 863-61-6
NAD free acid	Sigma-Aldrich	NAD100-RO; CAS 53-84-9
β-NMN	Sigma-Aldrich	N3501; CAS 1094-61-7
Metformin	Sigma-Aldrich	D150959; CAS 1115-70-4
Antimycin A	Sigma-Aldrich	A8674; CAS 1397-84-0
Sodium fluoride	Sigma-Aldrich	S7920; CAS 7681-49-4
Sodium Lactate, L-[2-3H], 10-20Ci/mmol	American Radiolabeled Chemicals	ART 0430
Resazurin	Sigma-Aldrich	R7017; CAS 62758-13-8
Critical Commercial Assays		
Arkray Lactate Pro 2 lactate test meter	ArkRay	LT-1730
pHrodo Red AM Intracellular pH Indicator	Thermo Fisher Scientific	P35372
NAD+/NADH Quantification Colorimetric kit	BioVision	K337-100
CellTiterGlo Luminescent Assay	Promega	G7570
Lactate-Glo Luminescent Assay	Promega	J5022
Experimental Models: Cell Lines		
HAP1 wild-type	Horizon Discovery	wt line C631
HAP1 MCT1-KO cl2	Horizon Discovery	HZGHC002882c002
HAP1 MCT1-KO cl10	Horizon Discovery	HZGHC002882c010
HAP1 MCT4-KO cl1	Horizon Discovery	HZGHC001844c001
HAP1 MCT4-KO cl10	Horizon Discovery	HZGHC001844c010
HeLa	ATCC	CCL-2
HCT116	ATCC	CCL-247
HL60	DSMZ	ACC-3
K562	DSMZ	ACC-10
SkBr3	Laboratory of Nancy Hynes, FMI, Basel	N/A
MDA-MD-453	Laboratory of Nancy Hynes, FMI, Basel	N/A

(*Continued on next page*)

Continued		
REAGENT or RESOURCE	SOURCE	IDENTIFIER
Experimental Models: Organisms/Strains		
Mouse: Male, 20 week old Liver-specific *Tsc1/Pten* double knock-out in mixed C57BL/6J,129/SvJae,BALB/cJ background.	Michael Hall Laboratory (Hindupur et al., 2018)	N/A
Software and Algorithms		
Prism 7	GraphPad Software	Version 7.0d

CONTACT FOR REAGENT AND RESOURCE SHARING

Further information and requests for resources and reagents should be directed to and will be fulfilled by the Lead Contact, Prof. Michael N. Hall (m.hall@unibas.ch).

EXPERIMENTAL MODEL AND SUBJECT DETAILS

Mouse Liver tumor model

Liver-specific *Tsc1* and *Pten* double-knockout mice were obtained by crossing *Tsc1*$^{lox/lox}$ mice (exons 17 and 18) with *Pten*$^{lox/lox}$ mice (exons 4 and 5) to transgenic mice expressing Cre recombinase under the control of the hepatocyte-specific albumin promoter (Alb-CreTg/0) to generate liver-specific double-knockout (*Tsc1*$^{lox/lox}$*Pten*$^{lox/lox}$ Alb-CreTg/0) mice. The mice produced were on mixed genetic background (C57BL/6J, 129/SvJae, BALB/cJ). Mice were housed under temperature and humidity-controlled conditions, in a 12-h light/dark cycle with lights switched on between 0600 to 1800. All experiments were conducted on 20-week old male mice. In all experiments, mice were fasted overnight before euthanasia by CO_2 inhalation.

METHOD DETAILS

Cell proliferation assays

96-well plates were plated at densities of 3000 cells/150 µL medium (suspension cells) or 2000 cells/150 µL medium (adherent cells) and growth measured after 3 (suspension cultures) or 5 days (adherent cell lines) by addition of 0.1vol resazurin (final concentration 50 µM). The plates were incubated for several hours for resazurin conversion before fluorescent reading (535/595nm Ex/Em) with a plate reader. After background subtraction with the medium only control, growth curves were either plotted using non-normalized data, or was normalized to untreated control cells and expressed as percentages. Each data point was performed in triplicate.

Immunoblotting

Cells were lysed in RIPA buffer containing protease and phosphatase inhibitors and total protein (20-40 µg) resolved on SDS-PAGE gels. Proteins were transferred onto nitrocellulose membranes for immunoblotting. Signals were detected with HRP-conjugated secondary antibodies and visualized with ECL detection reagent (GE Healthcare).

Co-immunoprecipitation

Cells were lysed in CHAPs buffer (120mM NaCl, 40mM HEPES pH 7.4, 50mM NaF, 1mM EDTA, 10mM β-glycerophosphate, 0.3% CHAPS, supplemented with protease and phosphatase inhibitors). Cell lysate was pre-cleared by spinning at 20000rpm, 4°C, 20 minutes followed by rotation with protein A/G beads for 1 hour at 4°C. 5 µg of rabbit anti-CD147 antibody (Abcam ab6416) was added to cleared lysates and rotated overnight at 4°C. Immune complexes were pulled down with protein A/G beads and washed 3x with 1ml CHAPS buffer. Proteins were eluted with 0.1M glycine (pH 3) and resolved on SDS-PAGE gels. Target proteins were detected by immunoblotting with the following mouse monoclonal antibodies: CD147 (R&D MB972), MCT1 (Santa Cruz sc365501) and MCT4 (Santa Cruz sc376140).

DARTS assay

HCT116 cells were grown to 80% confluency in DMEM and pre-treated with compounds (10 µM) for 2 hours. After washing with PBS, cytosolic depletion was performed by scraping cells and incubating with 1.9ml digitonin buffer (digitonin 0.01%, NaCl 150mM, HEPES 50mM pH 7.4) for 30 minutes on ice before centrifugation (2000 g, 10 minutes, 4°C). Cell pellets were solubilized with 400 µL lauryl maltoside buffer (lauryl maltoside 1%, NaCl 150mM, HEPES 50mM pH 7.4) for 3 hours on ice and centrifuged at 10000 g, 10 minutes, 4°C. Protein concentration was adjusted to 2mg/ml protein with lauryl maltoside buffer and treated with compounds (10 µM) overnight on ice. Digestion with thermolysin (Roche, P1512) was performed in a V-bottom 96-well plate (1ng thermolysin/µg protein) with shaking for 30 minutes at 37°C. Digestion was stopped by addition of 2x SDS-PAGE buffer and samples

were resolved on SDS-PAGE gels and probed for MCT1 (Santa Cruz, sc-50324), MCT4 (Santa Cruz, sc-50329), CD147 (Santa Cruz, sc-13976) and β-1 integrin (Santa Cruz, sc-374430).

Mouse experiments

Animal experiments were performed in accordance with the federal guidelines for animal experimentation and were approved by the Kantonales Veterinäramt of Kanton Basel-Stadt. Mice were injected intra-peritoneally with syrosingopine (7.5mg/kg body weight) 16 hours and 1 hour before sacrifice. Mice were euthanized with CO_2 and blood taken from the body cavity for lactate measurement. Serum lactate levels were measured using an Arkray Lactate Pro 2 lactate test meter with corresponding test strips. Intracellular lactate was measured in liver tumor nodules. Nodules were excised (3 per mouse) and ground to a fine powder in liquid nitrogen. Pulverized tumor material was resuspended in 20 μL water and freeze-thawed 3 times (dry-ice/37° water bath) to release cell contents. Lactate was measured with the lactate test meter. Protein concentration was measured by BCA to normalize the lactate measurements between the nodules.

ATP and lactate measurement

ATP levels were measured by lysing cells with CellTiterGlo reagent (Promega) and measuring released luminescence with a luminometer. Experiments were set up in 96-well format (variable cell numbers/100 μL medium) and at the desired time-point, ATP content was determined with 100 μL CellTiterGlo Reagent.

Lactate levels were measured enzymatically in 96-well plates according to manufacturer's specifications (Lactate-Glo Assay, Promega, J5022). 15000 cells/well or 30000 cells/well were seeded respectively for HeLa and HAP1. After compound incubation, cells were washed with PBS and lysed with 22.5 μL HCl (0.2N). Cell lysates were neutralized with 7.5 μL 1M Tris-base and incubated with 30 μL detection reagent. Luminescence was recorded after 1 hour and intracellular lactate concentrations determined from a standard curve. Extracellular lactate production was measured in the medium with background subtraction from fresh medium. Lactate levels were normalized to protein content (Pierce BCA, 23225) from duplicate plates after lysing cells with NP40 (1% v/v). Alternatively, lactate levels were also measured using an Arkray Lactate Pro 2 lactate test meter with corresponding test strips. Extracellular lactate was measured directly from culture medium. 500,000 cells were seeded in 2ml medium and incubated for 6 hours with drug treatments as indicated. Intracellular lactate was measured from the cell pellet. Cells were spun down by touch-spin and resuspended in 20 μL dH_2O. The pellet was lysed by freeze-thawing 3x in a dry-ice/water bath and the released lactate measured.

Intracellular pH measurement

Intracellular pH was determined with pHrodo Red AM (Thermo Fisher Scientific, P35372). HeLa cells grown in DMEM (FCS 10%) were seeded in 96-well plates (2000 cells/well) and medium replaced after 24 hours with DMEM buffered with HEPES 20mM pH 7.0-7.6 (Sigma), FCS 1%. After drug treatment, cells were washed with HBSS, HEPES 20mM and labeled with pHrodo Red AM dye for 30 minutes. After washing with HBSS, HEPES 20mM, cell fluorescence was measured (560/585 Ex/Em) and intracellular pH determined according to calibration standards (Thermo Fisher Scientific, P35379).

Radiolabeled lactate uptake and export assays

Lactate export: Cells were counted and seeded at a density of $2x10^6$ cells per ml in RPMI1640 medium. Medium was adjusted to pH6 to favor uptake of exogenous lactate, particularly for MCT1-KO cells. Radioactive L-lactate (American Radiolabeled Chemicals, ART0430, 1mCi/ml) was added (2 μCi per sample) to cells at 37° for 60 minutes. Drugs were added for the final 30 minutes of labeling. Based on pilot experiments which showed a rapid initial efflux of labeled lactate after washing the cells, a drug pre-treatment of 30 minutes was held to be optimal to allow time for drug-mediated inhibition before performing the lactate export assays. At the end of pulse labeling (60 minutes), cells were spun down (2000rpm, 5 minutes), washed once with 1ml ice-cold RPMI1640 medium (pH 6) and resuspended in fresh ice-cold, label-free RPMI1640 medium (pH 7.4 and containing the respective drug according to the experiment) and kept on ice. At appropriate time points, the cell suspension was sampled ($2x10^6$ cells per sampling). Cells were quickly pelleted by touch-spin and separated into cell pellet and supernatant. The pellet was lysed with 500 μL 0.1M HCl and radioactivity in 450 μL of lysed pellet measured in 10ml of scintillation fluid in a scintillation counter to determine the amount of radioactivity retained in the cell. Radioactivity in the supernatant was also counted by scintillation counting to determine how much radioactivity was exported out of the cells into the supernatant over time.

Lactate uptake: Cells were labeled and drug-treated similar to the export assay. At the end of the pulse/drug-treatment (60/30 minutes), cells were spun down (2000rpm 5 minutes), briefly rinsed and the cell pellet was lysed with 500 μL 0.1M HCl. Radioactivity in 450 μL of lysed pellet was measured in 10ml of scintillation fluid in a scintillation counter to determine the amount of radioactivity present in the cell. As the transporters work in both directions, measuring the amount of radioactivity present in the cell after a 60 minute pulse shows the equilibrium reached between lactate import and export.

NAD+/NADH measurements

Total NAD (NADt) and NADH levels were measured using a NAD/NADH Quantitation Colorimetric Kit (Biovision). Cells were seeded (3-$5x10^5$ cells/5ml in full medium. At the end of drug treatment period, cells were harvested and the pellets processed according to the kit instructions. NADt (NAD1 and NADH) levels were measured were read at OD 450nm. NADH was determined

by decomposing NAD+ at 60°, 30 mins before performing detection reaction. NADt and NADH readings were normalized to protein content of the cell lysate. NAD+/NADH ratio was determined from the measured NADt and NADH values by the following formula: (NADt-NADH)/NADH.

QUANTIFICATION AND STATISTICAL ANALYSIS

Statistical analysis was performed using Prism7 (ver 7.0d, GraphPad software). Statistical tests of data were either two-tailed unpaired Mann-Whitney test (Figures 1A and 1B), or two-tailed unpaired Students's 't'-test (all other analyses). Replicate information is indicated in the figure legends.

Cell Reports, Volume *25*

Supplemental Information

Dual Inhibition of the Lactate Transporters

MCT1 and MCT4 Is Synthetic Lethal with Metformin

due to NAD+ Depletion in Cancer Cells

Don Benjamin, Dimitri Robay, Sravanth K. Hindupur, Jens Pohlmann, Marco Colombi, Mahmoud Y. El-Shemerly, Sauveur-Michel Maira, Christoph Moroni, Heidi A. Lane, and Michael N. Hall

Figure S1

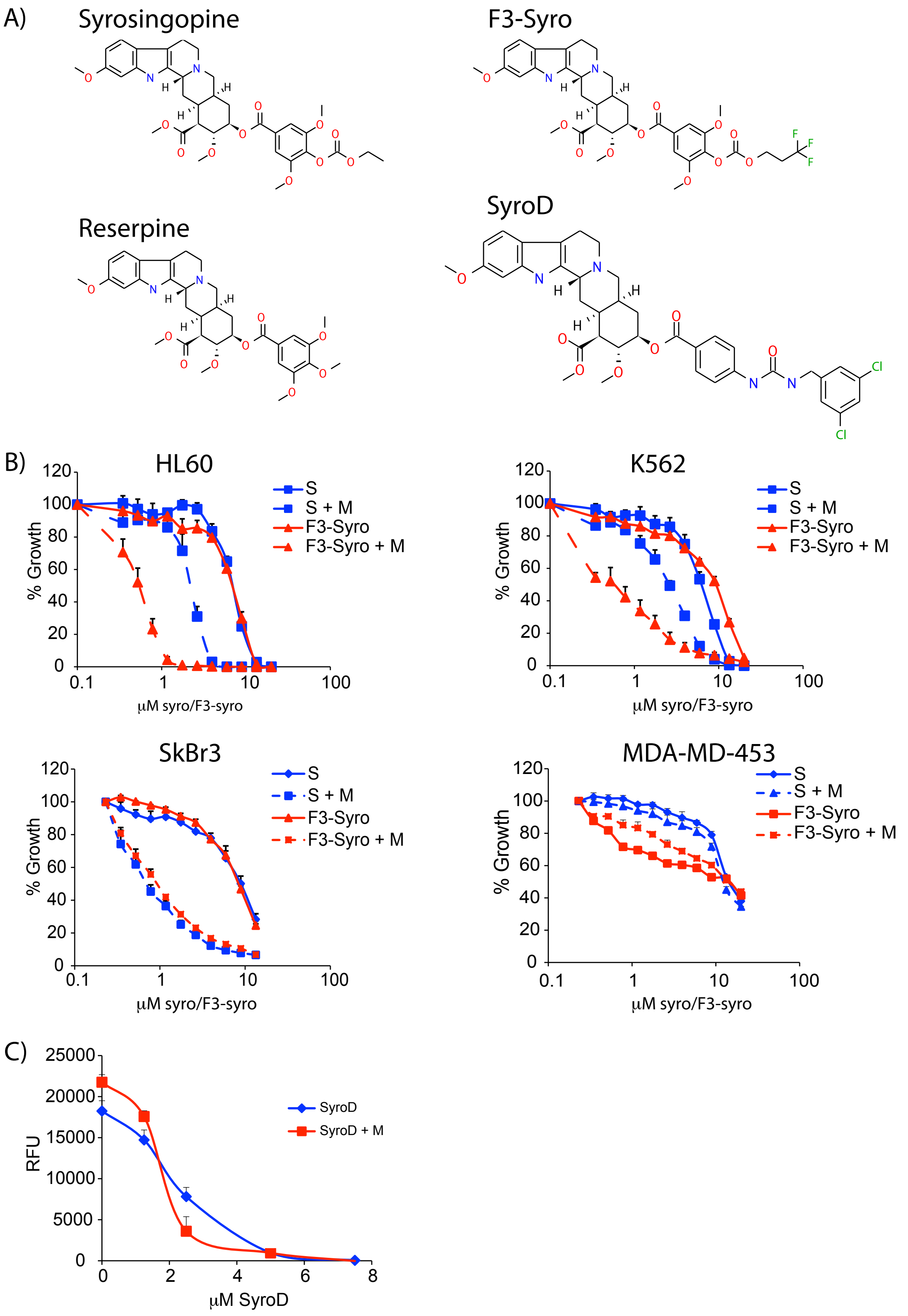

Figure S1. Relative potency of syrosingopine derivatives for synthetic lethality with metformin. Related to Figures 1 and 2. (A) Structures of the parent compound reserpine and its derivatives syrosingopine and F3-syro. SyroD is inactive in eliciting synthetic lethality with metformin. **(B)** Synthetic lethality between syrosingopine (S) or F3-syro (F3-syro) and metformin (M) in the human cancer lines HL60, K562, SkBr3 and MDA-MB-453. **(C)** Proliferation of HL60 cells titrated with SyroD in the presence/absence of metformin. Survival was measured after 3 days of drug treatment. All data points were measured in triplicate, represented as mean +/- SEM.

Fig S2

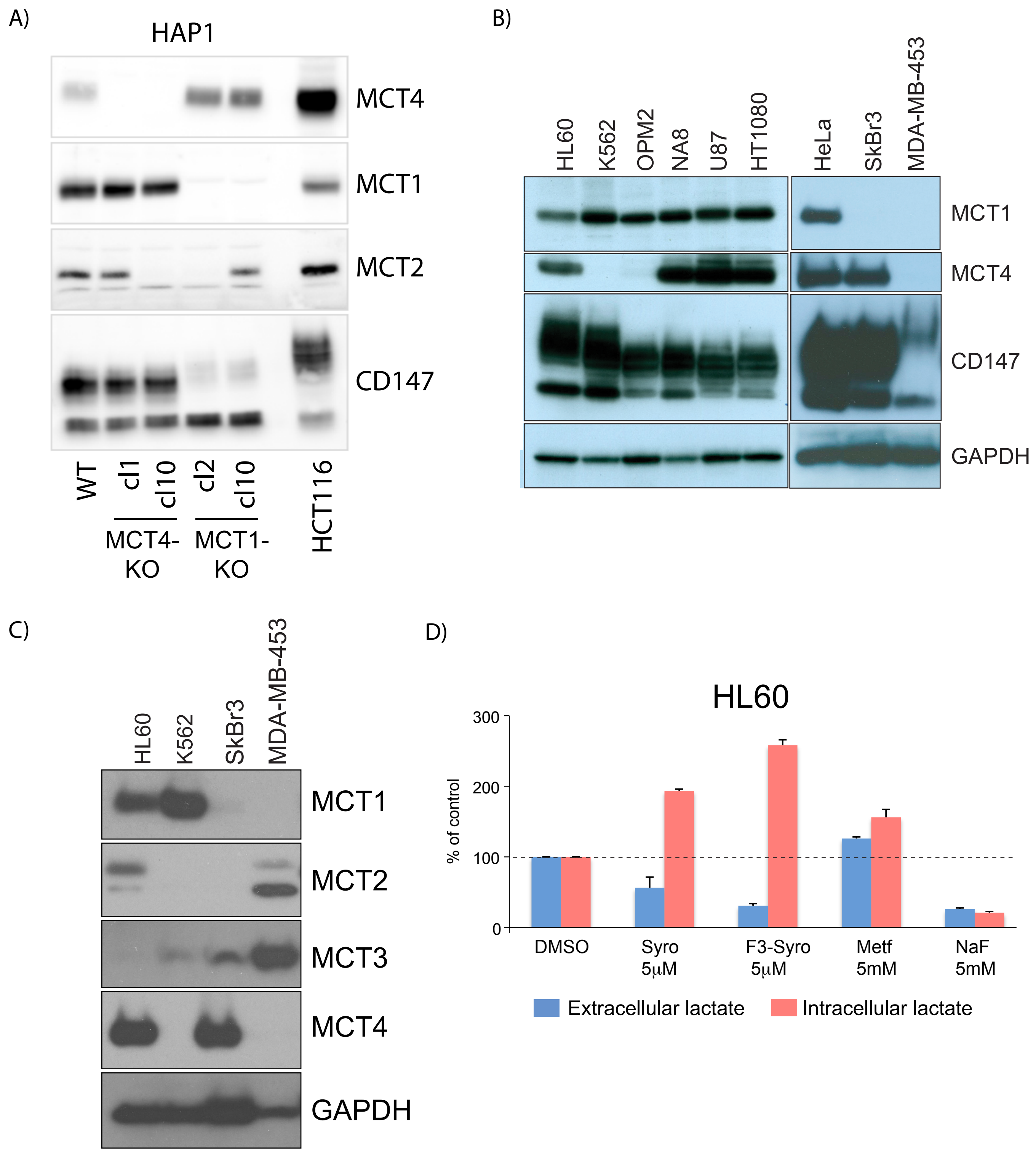

Figure S2. MCT isoform expression in HAP1 MCT-knockout panel and human cancer cell lines. Related to Figures 2, 3 and 5. (A) Immunoblot of HAP1 cells deleted for MCT1 (clones cl2 and cl10) or MCT4 (clones cl1 and cl10). **(B)** Immunoblot for MCT1 and MCT4 expression and their chaperone CD147 in various human cancer cell lines. HL60, K562, SkBr3 and MDA-MB-453 were selected to compose a cell panel. **(C)** Immunoblot of MCT1-4 in the cell panel. **(D)** Extra- and intra-cellular lactate levels in HL60 cells after 6 hours of treatment with syrosingopine, F3-syro, metformin and the glycolytic inhibitor NaF. All data points were measured in duplicate, bars are mean +/- SEM.

Figure S3

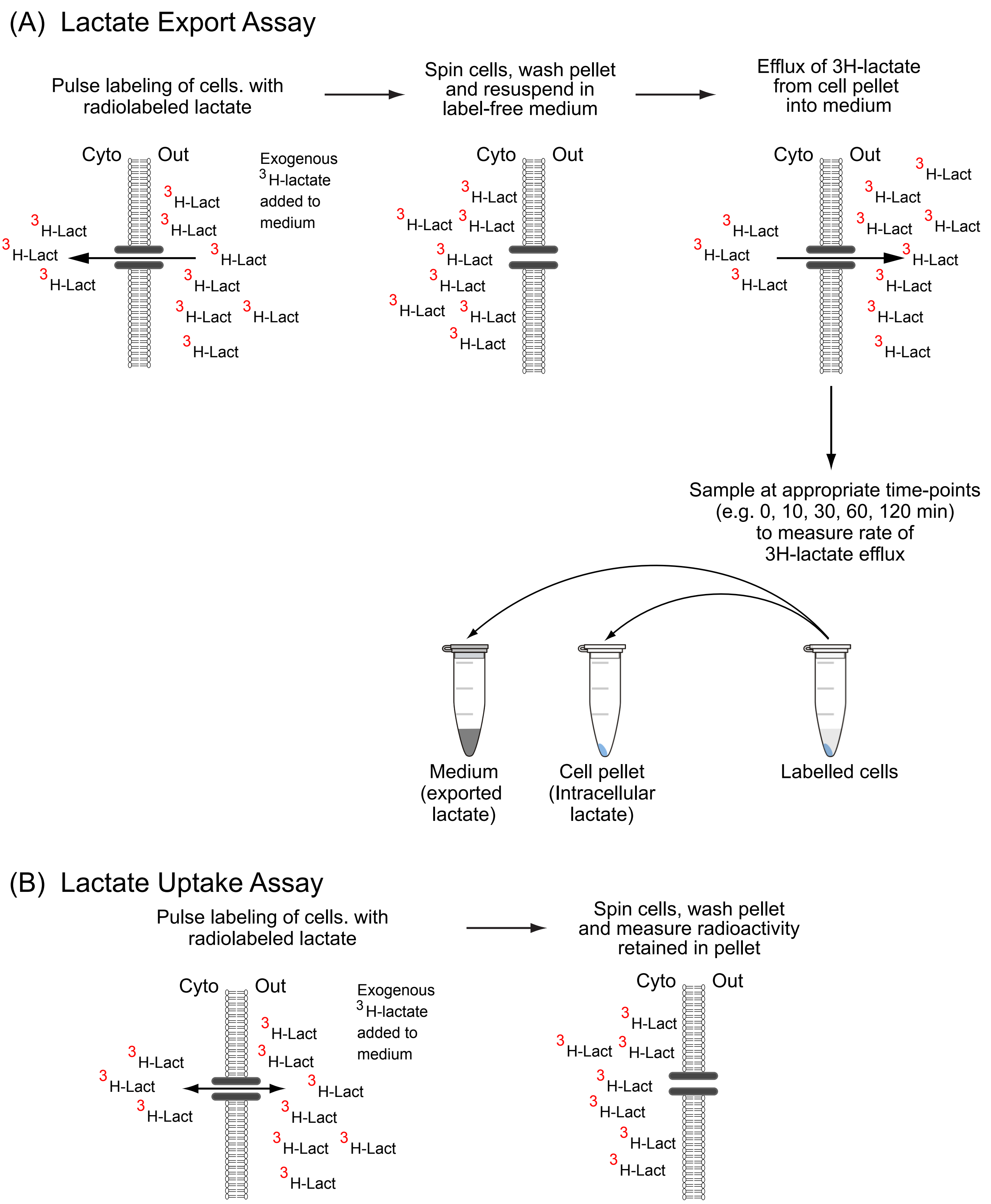

Figure S3. Schematic diagrams of radioactive lactate transport assays. Related to Figures 3 and S4. (A) lactate export assay and **(B)** lactate uptake assay.

Figure S4

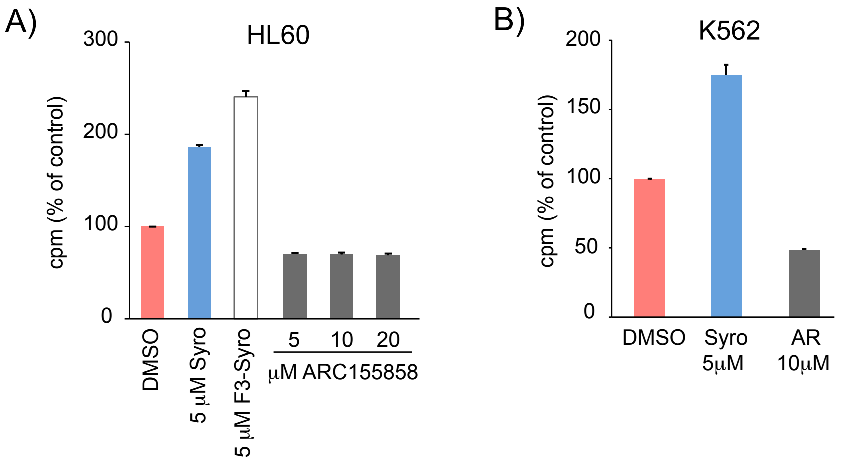

Figure S4. Radioactive lactate uptake and the effect of indicated drugs. Related to Figure 3. Radioactive lactate present in the cell pellet of **(A)** HL60 and **(B)** K562 cells. Labeling pulse was 1 hour. Each data point was measured in duplicate, bars are mean +/- SEM.

Figure S5

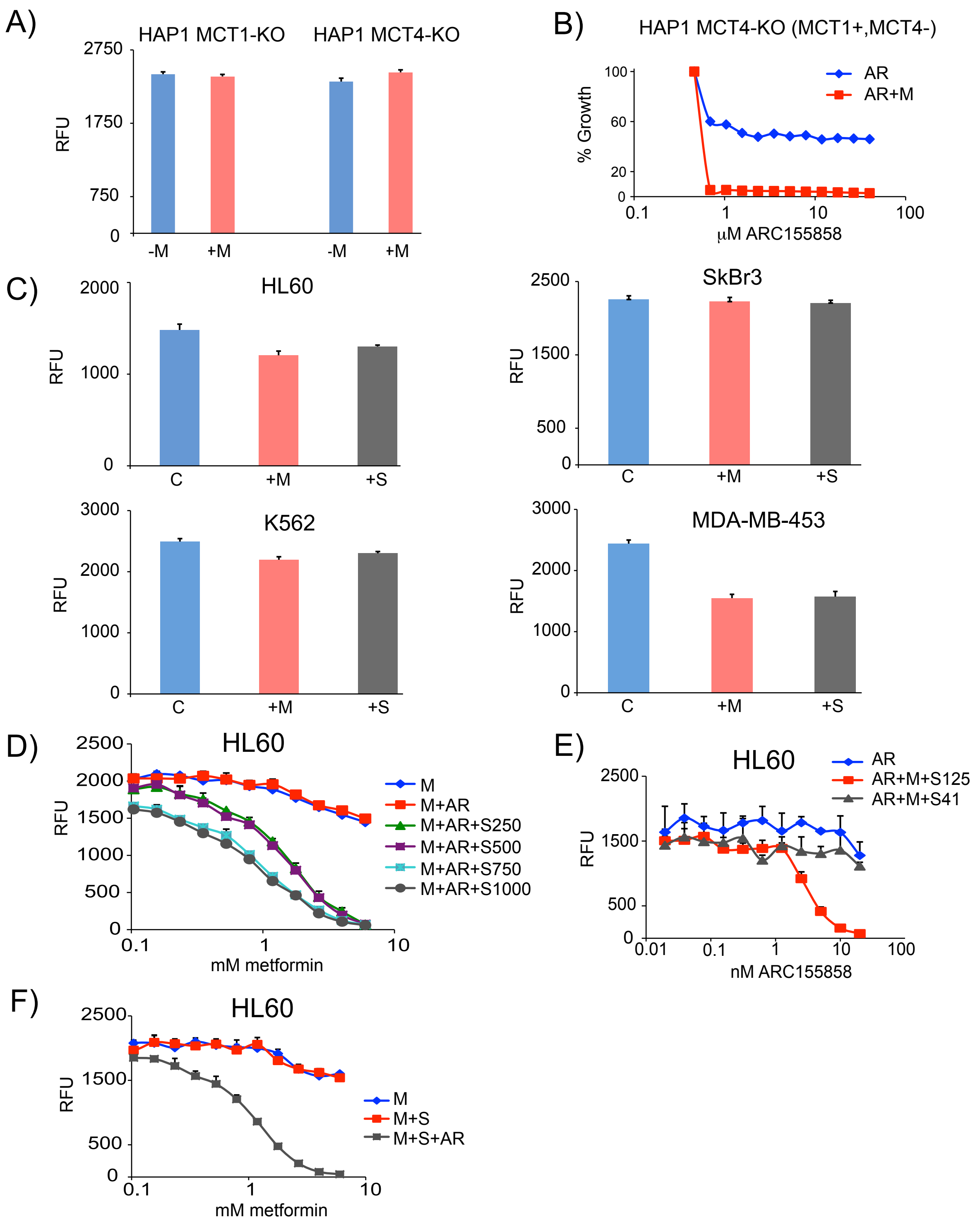

Figure S5. Survival curves of cell line models with varying combinations of metformin, syrosingopine and ARC155858. Related to Figure 5. (A) 4mM metformin (M) has no impact on proliferation in HAP1 MCT1-KO and MCT4-KO cells. **(B)** Survival curve of HAP1 MCT4-KO cells treated with the MCT1 inhibitor ARC155858 (AR), alone or in combination with 4mM metformin. **(C)** Effect of metformin (4mM) and syrosingopine (S, 0.5μM) on proliferation in HL60, K562, SkBr3 and MDA-MB-453 cells. **(D)** Triple drug combination in HL60. Cells were titrated with metformin alone or in a background of 20nM ARC155858 and syrosingopine at various concentrations (250, 500, 750, 1000nM). **(E)** HL60 cells treated with increasing concentrations of ARC155858 in the presence of metformin (4mM) and syrosingopine (41 and 125nM). **(F)** HL60 cells treated with increasing concentrations of metformin in the presence of syrosingopine (1μM) and ARC155858 (20nM). Growth measured after 3 days. Data points were measured in triplicate and show as mean +/- SEM. RFU = relative fluorescence units

Figure S6

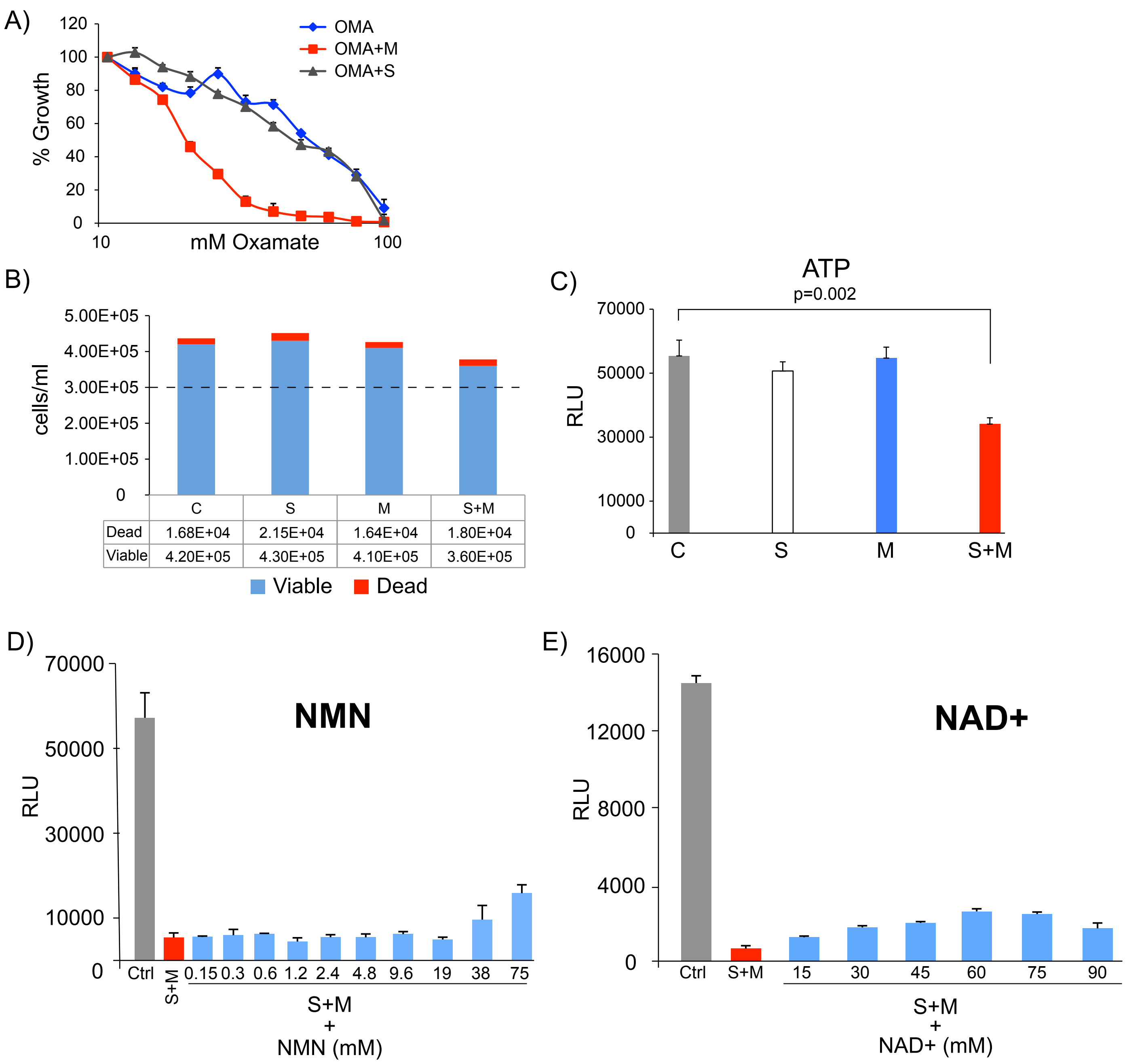

Figure S6. Syrosingopine-metformin treatment impacts NAD+/NADH metabolism. Related to Figure 6. (A) 3-day proliferation assay of HL60 cells titrated with the LDH inhibitor oxamic acid (OMA), and in combination with metformin (M, 4mM) or syrosingopine (S, 4μM). Data points were measured in triplicate, error bars are mean +/- SEM. **(B)** Cell viability counts of HL60 cells after metformin (4mM) or syrosingopine (5μM) treatment for 8 hours, slight growth retardation but no drop in viability was observed for S+M treated cells. Initial cell density shown in dotted line. **(C)** ATP levels of cells in (B) after normalisation for cell numbers. For (B,C) parallel experiments were conducted and data points measured in duplicate. **(D, E)** ATP levels in HL60 cells treated for 30 hours with 5μM syrosingopine and 4mM metformin (S+M). Increasing amounts of NMN or NAD+ were titrated in S+M treated cells. Data points measured in duplicate, bars are mean +/- SEM.
RLU = relative light units
